# A dopamine mechanism for reward maximization

**DOI:** 10.1073/pnas.2316658121

**Published:** 2024-05-08

**Authors:** Wolfram Schultz

**Affiliations:** ^a^Department of Physiology, Development and Neuroscience, University of Cambridge, Cambridge CB2 3DY, United Kingdom

**Keywords:** reward prediction error, reinforcement learning, prediction, iteration, recursion

## Abstract

Individual survival and evolutionary selection require biological organisms to maximize reward. Economic choice theories define the necessary and sufficient conditions, and neuronal signals of decision variables provide mechanistic explanations. Reinforcement learning (RL) formalisms use predictions, actions, and policies to maximize reward. Midbrain dopamine neurons code reward prediction errors (RPE) of subjective reward value suitable for RL. Electrical and optogenetic self-stimulation experiments demonstrate that monkeys and rodents repeat behaviors that result in dopamine excitation. Dopamine excitations reflect positive RPEs that increase reward predictions via RL; against increasing predictions, obtaining similar dopamine RPE signals again requires better rewards than before. The positive RPEs drive predictions higher again and thus advance a recursive reward-RPE-prediction iteration toward better and better rewards. Agents also avoid dopamine inhibitions that lower reward prediction via RL, which allows smaller rewards than before to elicit positive dopamine RPE signals and resume the iteration toward better rewards. In this way, dopamine RPE signals serve a causal mechanism that attracts agents via RL to the best rewards. The mechanism improves daily life and benefits evolutionary selection but may also induce restlessness and greed.

Successful evolutionary selection requires biological organisms to survive and stay healthy until they can propagate their genes into the next generation. Individual survival depends on getting nutrients and other substances from the environment. The substances are packaged in foods and drinks and constitute natural rewards. Only individuals who get the best rewards will make it through evolutionary selection (1–3), in particular during shortages, without necessarily being aware of the consequences of their actions. Thus, reward maximization is crucial for individual survival and evolutionary fitness. The rewards being maximized include non-natural rewards that benefit the individual human or animal agent, like money, but exclude substances of abuse that hijack biological reward-mechanisms without unequivocally assuring welfare and evolutionary fitness.

Given its importance, reward maximization is a key topic of behavioral theories. Economic theories define necessary and sufficient conditions for maximizing reward in choices (4, 5). Neurons in several brain structures signal decision variables underlying economic choices, including object value, action value, and chosen value (6–8) that may attract choices toward better rewards (9–11). Separately, reinforcement learning (RL) theory proposes formalisms by which agents learn predictions, actions, and policies that improve reward acquisition (12–16). Neurophysiological studies identify neuronal signals in dopamine neurons that report reward prediction errors (RPE) underlying RL (17). Thus, it has become interesting to understand how dopamine RPE signals might contribute to the acquisition and maximization of reward.

The idea of a neuronal involvement in an RL process of reward maximization goes back to Harry Klopf’s Hedonistic Neuron that maximizes its discharge activity as a local analog of pleasure (18). The idea became a testable hypothesis when the dopamine RPE signal was identified (17) and, in particular, when it became recognized that monkeys and rodents press levers and make choices that result in dopamine RPE-like signals (19–35). When naturally evoked, such dopamine RPE signals may engage a recursive neuronal RL mechanism that attracts agents iteratively to the maximal available rewards.

## Reinforcement learning

RL theories explain how biological agents obtain the best available rewards. Agents learn to increase the probability of behavior that leads to more reward and avoids less reward the next time around (positive and negative reinforcement, respectively) (14). Basic RL forms are Pavlovian conditioning that describes how an arbitrary stimulus becomes a reward predictor for an individual reward (12, 15) and temporal difference (TD) learning that is concerned with the total overall outcome of a series of rewarding events (13).

### Reward prediction error.

**Fig. 1.**
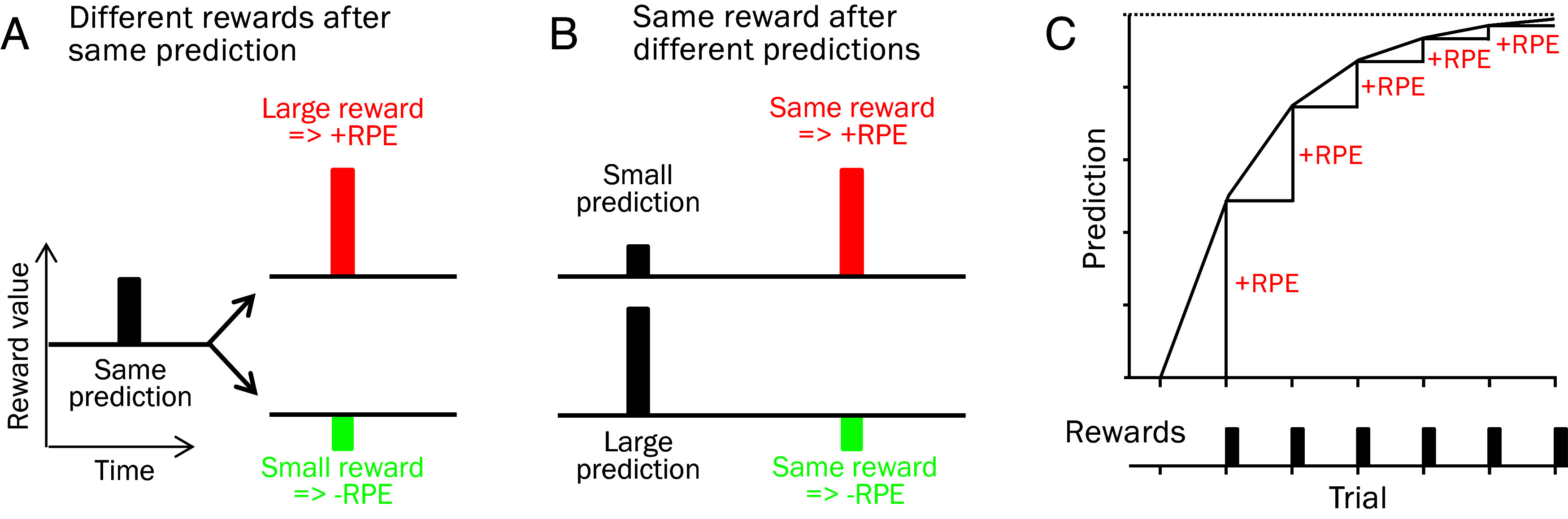
RPE in RL. RPE equals reward minus prediction. (*A*) Different rewards after the same prediction elicit different RPEs. (*B*) The same rewards after different predictions elicit different RPEs. (*C*) Asymptotic appetitive Pavlovian conditioning: Repeated delivery of constant rewards (*Bottom*) elicits positive RPEs that increase the reward prediction. With increasing prediction, RPEs decrease gradually over trials (decreasing RPE = constant reward – increasing prediction).

The critical variable for RL is the RPE (15, 16), defined as value difference between received reward and predicted reward. Compared to a constant prediction, a larger reward generates a positive RPE, a smaller reward generates a negative RPE, and no difference fails to generate any RPE (Fig. 1*A*) (thus, RPEs are not limited to performance errors). Importantly, against different predictions, the same reward elicits a positive RPE after a small prediction but a negative RPE after a large prediction (Fig. 1*B*). Hence, the advance information provided by a prediction constitutes a reference for the subsequent reward and is a key factor determining the RPE.

According to general RL principles, positive RPEs provide positive reinforcement and negative RPEs provide negative reinforcement. In Pavlovian conditioning, RPEs generate and update the prediction of the reinforcer (15). Thus, a positive RPE from a better-than-predicted reward increases the reward prediction, a negative RPE from a worse-than-predicted reward decreases the reward prediction, and a fully predicted reward failing to induce an RPE does not affect the reward prediction. By providing positive or negative reinforcement, RPEs strengthen behavior that respectively increases or decreases the probability of receiving the same reward again.

### TD Learning.

The crucial variable for reward maximization is not the individual reward but the total outcome achieved in the long run This insight is captured in TD RL in which a series of reward predictors is updated to maximize the total outcome, called "state value" (as opposed to individual "reward") and defined as discounted sum of all individual primary and higher-order rewards (13). The RPE in TD is the temporal value difference between total experienced outcome and current prediction at each point in time: finite value difference divided by finite TD between prediction and reward (or next reward-predicting stimulus) (Δv/Δt; v for reward value, t for time) or differential temporal value change dv/dt (first derivative of value over time). Thus, the TD RPE is an RPE in time, representing both value difference and time difference between reward and prediction.

### Recursion in RL.

In the most straightforward case of model-free appetitive Pavlovian RL (Fig. 1*C*), the repeated occurrence of the same unpredicted reward elicits RPEs that drive the reward prediction toward an asymptote. As the prediction increases, the reward elicits gradually decreasing RPEs that approach zero as the prediction asymptotes (Fig. 1*C* red). Thus, the RPE is a part of a recursive process: The RPE updates the prediction that determines the next RPE which then updates the prediction again that determines the following RPE. With asymptotic RL, the iteration continues until the RPE has reached zero. In TD learning, the recursion involves every event.

### Conclusions.

RL engages a recursion between reward, RPE and prediction: An RPE updates a prediction, the updated prediction determines the next RPE, which then updates the prediction again. The process iterates until no further better or worse rewards occur to generate RPEs. The recursion is an important mechanism for the proposed reward maximization.

## The Dopamine RPE Signal

The fast phasic responses of dopamine neurons to rewards and reward-predicting stimuli depend on the unpredictability of these events, irrespective of Pavlovian, TD, or operant scenarios (36, 37). Specifically, dopamine neurons respond with short excitations to better-than-predicted rewards that elicit positive RPEs, they show slightly longer inhibitions following worse-than-predicted rewards that elicit negative RPEs, and they don’t respond to rewards that occur as predicted and fail to elicit RPEs. Dopamine inhibitions occur also with negative RPEs elicited by negatively valued events, such as punishers (38). Thus, against a prediction of small reward, a better reward elicits a positive RPE and dopamine excitation, but against a prediction of large reward, the same reward elicits a negative RPE and dopamine inhibition (Fig. 1*B*; 39). This characteristic of the dopamine RPE signal is a critical feature of the proposed reward maximization mechanism that builds on the effects of dopamine RPE signals on behavior.

### TD Prediction Error Signal.

The dopamine RPE response corresponds to key features of TD RL (17, 40, 41). They occur not only to ultimate rewards but also to single and sequential reward-predicting stimuli (17, 41–48) and do not differ categorically from responses to ultimate rewards (other than reduction by temporal discounting). The slopes of positive and negative RPE responses vary across individual dopamine neurons in correspondence to efficient distributional TD RL versions (49). In keeping with the temporal aspects of TD RL, the dopamine RPE signal is time sensitive and occurs even when the laboratory provides an overall reward prediction context. Rewards occurring at unpredicted moments, earlier rewards than predicted, and rewards at inaccurately perceived temporal reward predictions elicit positive temporal RPEs and dopamine excitations (37, 50, 51); delayed rewards lead to negative temporal RPEs and dopamine inhibitions at the original time and to positive temporal RPEs and dopamine excitations at the later time. Thus, dopamine neurons code time-specific RPEs at every rewarding event, namely temporal value difference Δv/Δt between prediction and reward, or differential temporal value change dv/dt. Further, small dopamine RPE signals backpropagate from reward to stimuli in some learning tasks (52), as stipulated by major TD RL models (17, 40). With these characteristics, the dopamine RPE signal could serve to update reward predictions according to basic assumptions of TD RL (13).

### Dopamine Diversity.

**Fig. 2.**
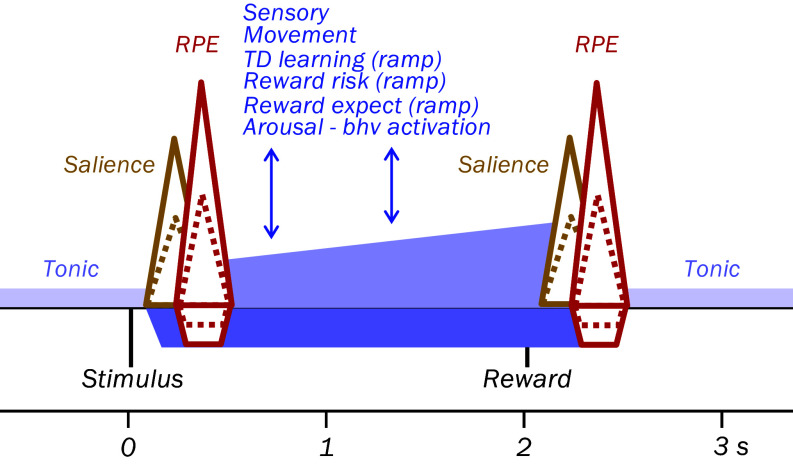
Diverse dopamine signals. Brown: Unidirectional salience response to any stimulus. Red: Bidirectional RPE response to reward-predicting stimuli and ultimate rewards. Dotted lines indicate variation depending on prediction. Blue: Slower and lower excitations and inhibitions to various events (TD, temporal difference; bhv, behavior). Light blue: Tonic or slowly changing background dopamine activity enabling movement and cognition.

Dopamine neurons show also an early salience response whose unidirectional excitatory nature distinguishes it from the subsequent bidirectional RPE signal (53) (Fig. 2). Besides these rapid signals, slower dopamine changes report stimuli, movements, small backpropagating TD RPEs, reward risk, reward expectation, general arousal, and behavioral activation. In addition, dopamine neurons show tonic or slowly modulating background activity that provides extracellular dopamine concentrations and possibly affects the efficacy of phasic dopamine signals on dopamine receptors. The diversity adds to the complexity of dopamine functions and refutes the notion of "one brain structure equates one function", without challenging the proposed reward maximizing function of dopamine RPE signals. For details, see *SI Appendix*, *SI Text 1* and Fig. S1.

### Conclusions.

The phasic dopamine response to rewards and reward-predicting stimuli constitutes the fastest and strongest dopamine change and codes RPEs according to RL and TD RL theories. This signal is the key component of the proposed reward maximization mechanism.

## Subjective Reward Value

The value of a reward depends on the benefits it provides for an agent. Therefore, reward value is subjective and does not completely derive from physical reward characteristics. Hence, agents should maximize subjective value, not objective value, and meaningful neuronal signals for reward maximization should reflect the subjective reward nature.

### Estimation of Subjective Value.

**Fig. 3.**
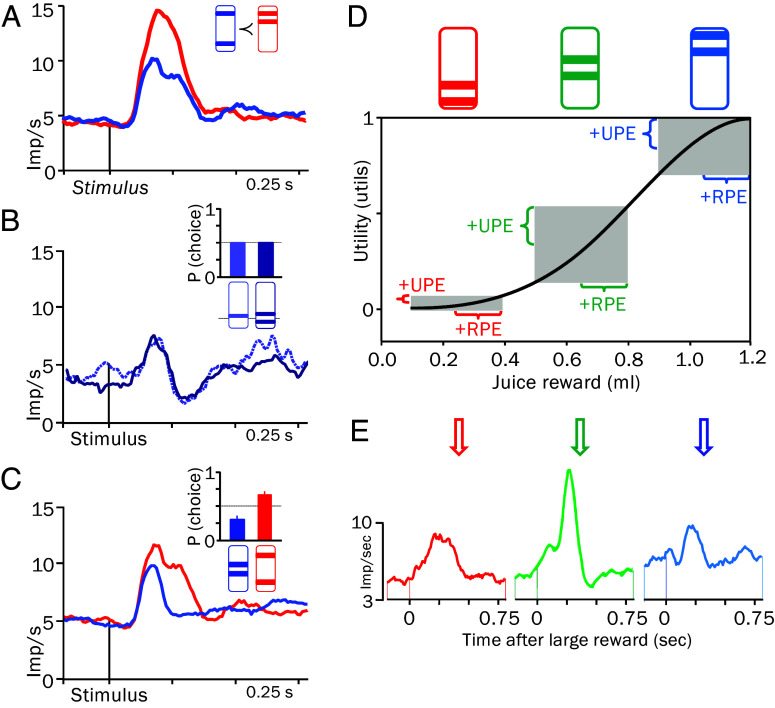
Subjective reward value tests. (*A*) Behavioral preference and stronger dopamine response to gamble-predicting stimuli for better compared to worse gamble (red vs. blue; equiprobable rewards: *P* = 0.5 each reward). (*Inset*) Bar stimuli (higher bar indicates more juice reward, ≻ indicates preference). (*B*) Choice indifference and similar dopamine response. (*Inset*) Choice indifference [*P* (choice each option) = 0.5] between safe reward (single bar; reward *P* = 1.0) and gamble (*P* = 0.5 each reward). (*C*) Increased subjective value due to risk despite the same mean: behavioral preference and stronger dopamine response to stimuli predicting riskier gamble (red) compared to less risky gamble (blue). (*Inset*) Higher choice probability for riskier (red) than less risky (blue) gamble with the same mean amount. Occasional dominated choices (blue) reflect choice stochasticity. (*D*) Nonlinear utility function (black) transforms identical physical RPE into varying utility prediction errors (UPE: utility of received reward minus predicted mean utility). Three equiprobable gambles (*P* = 0.5) with the same difference between *Top* and *Bottom* reward amounts but different means. The same positive RPEs (0.15 mL) elicited by top gamble rewards (horizontal brackets) but different +UPEs due to nonlinear utility function (vertical brackets). (*E*) Variation of dopamine UPE response with steepness of utility function (green vs. red and blue), despite the same physical RPE. Image credit: Panels *A*, *C*, and *E* reproduced, which is licensed under CC BY 3.0, and panels *B* and *D* newly created, from own work (56). For details, see *SI Appendix*, *SI Text 2* and Fig. S2.

Subjective reward value can be estimated from probabilistic choices (4). To be meaningful, choices should follow first-order stochastic dominance, defined as follows: Every probabilistic option is at least as good as its alternative but better in at least one instance. In a test, each of two choice options has two equiprobable rewards, one of which is higher in one option (Fig. 3*A*). Indeed, monkeys prefer the better option, and their dopamine responses follow suit. Then, the psychophysically estimated choice indifference between safe and risky rewards serves to distinguish between subjective and objective value (Fig. 3*B*). At choice indifference, both options have equal subjective value. However, the safe reward exceeds the mean objective value of the risky option, which demonstrates the subjective value gain by risk. Importantly, by choosing the objectively smaller risky reward on half the trials, the animal’s choices reflect subjective value, and dopamine responses are similar to both options. More formal assessments of subjective value test second-order stochastic dominance where the riskier gamble "dominates" the choice (54) (Fig. 3*C*). The observed preference for the riskier option indicates higher subjective value, and dopamine neurons respond more to that better option. Further, monkeys’ choices satisfy three utility axioms (4, 55): i) completeness (either one or the other option is preferred or both options are equally preferred); ii) transitivity (if A is preferred to B, and B is preferred to C, then A is preferred to C); and iii) continuity (smooth probability-amount trade-off). Together, these tests demonstrate that subjective value is the key decision variable that is being maximized in economic choices.

Besides being synonymous with general subjective value, utility can be more stringently formalized as a mathematical function of objective reward amount. It can be estimated from risky choices between an adjustable safe reward and a preset option with two equiprobable rewards, using the so-called fractile or chaining procedure (57, 58) (for details, see *SI Appendix, SI Text 2*). The choices are fit by spline, power, logarithmic, negative-exponential, or multiparameter functions. Utility functions in monkeys are often S-shaped, gradually steepening with increasing reward amounts, then becoming more linear and flattening gradually with larger rewards (black curve in Fig. 3*D*) (56).

A utility function translates an objective RPE into a subjective utility prediction error (UPE) (Fig. 3*D*). Delivery of the larger reward of a binary equiprobable gamble elicits a positive RPE, and delivery of the smaller reward elicits a negative RPE. In analogy to RPEs, a UPE is the difference between the utility of the received reward and the expected (mean) utility. Importantly, the nonlinearity of the utility function transforms physically identical RPEs (horizontal) into different UPEs (vertical). Thus, rewards from gambles at steeper parts of the utility function elicit larger UPEs than gambles at flatter parts (Fig. 3*D*). Now we have a well-defined measure of subjective value that allows us to more precisely characterize the reward signal of dopamine neurons.

### Dopamine Coding of Subjective Reward Value.

Earlier work using reward types, risky choice, and temporal discounting suggested that dopamine neurons code reward amounts subjectively (59–61). When tested with formal utility functions, dopamine excitations elicited by identical positive objective RPEs are larger at the steeper central part of the utility function compared to its flatter peripheral parts (Fig. 3*E*). Such differential coding cannot be explained by identical RPEs and suggests coding of subjective reward value as UPEs (56) (reflecting varying marginal utility that historically defines nonlinear utility). Dopamine utility coding applies also to TD RL, where the dopamine signal for temporal reward difference (Δr/Δt or dr/dt) becomes a neuronal signal of temporal utility difference (Δu/Δt or du/dt).

### Conclusions.

The phasic dopamine signal concerns reward, its form is prediction error, and its metric is subjective reward value. The dopamine coding of subjective reward value is inferred from meaningful choices that comply with basic economic concepts. By coding subjective rather than objective reward value, the dopamine signals may directly participate in maximizing subjective reward value. We now need to determine how such dopamine signals may affect behavior.

## Behavioral Effects of Dopamine RPE Signal

### Attraction and Learning.

**Fig. 4.**
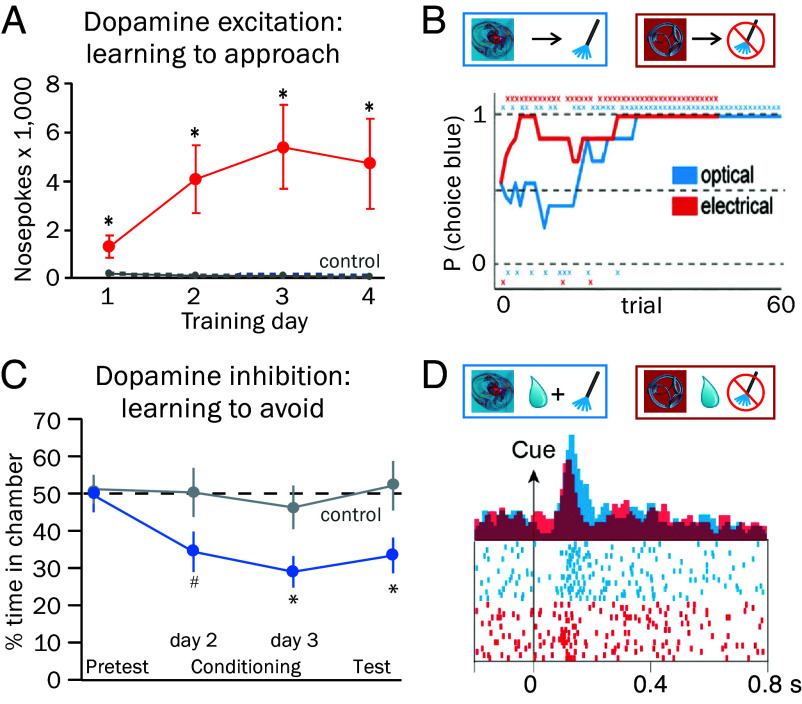
Effects of artificial dopamine neuron excitation and inhibition. (*A*) Mouse nosepoking following optogenetic dopamine excitation (±SEM). (*B*) Monkey choice preference for option with optogenetic (blue) or electric dopamine excitation (red) (red cue: no stimulation). (*C*) Mouse chamber avoidance following transsynaptic optogenetic dopamine inhibition. (*D*) Stronger monkey dopamine neuron response with optogenetic dopamine excitation added to juice reward (blue), compared to reward alone (red). Image credit: Panels *A* and *C* reproduced with permission from Elsevier Inc. (23, 26), and panels *B* and *D* reproduced from own work (35), which is licensed under CC BY 4.0.

Classic self-stimulation experiments demonstrate that rats repeatedly perform actions to touch levers that deliver electrical shocks to their brain (19). The animals’ intense attraction to the shock-delivering lever gave rise to the notion of activating the brain’s pleasure centers. Many effective brain sites are associated with midbrain dopamine cell bodies and striatal dopamine axons (20, 21), which is now confirmed by dopamine-specific optogenetic stimulation. Thus, the excitations of dopamine neurons make monkeys and rodents repeat lever pressing, approach behavior and choice (22–35) (Fig. 4 *A* and *B*). Opposite to excitation, optogenetically induced direct or transsynaptic inhibition of dopamine neurons increases behaviors in rodents that result in less such dopamine inhibition (Fig. 4*C*) (26, 28, 48, 62). These observations suggest a teaching function of artificially elicited dopamine excitations and inhibitions compatible with RL.

Besides these behavioral effects, artificial dopamine excitations induce responses of dopamine neurons to stimuli preceding the excitation (Fig. 4*D*) (31, 35). Once these reward predictions and dopamine responses to reward-predicting stimuli are established, omission of the artificial dopamine excitation elicits dopamine inhibition at the time at which the excitation would have occurred (31), demonstrating violation of an established reward prediction for this point in time similar to omission of natural rewards (50). Thus, the teaching function of dopamine excitation extends from behavior to neuronal signals. The artificial dopamine excitation seems to mimic a positive dopamine RPE signal that engages RL.

To extrapolate from these empirical findings, dopamine excitations and inhibitions may affect earlier predictions via TD RL. Any dopamine excitation would lead to predictive dopamine responses that propagate stepwise back to the earliest stimulus, and dopamine inhibitions would have analogous inhibitory effects. After learning is completed and no further RPEs occur, the dopamine response would have moved to the earliest predictive stimulus.

### Underlying Neuronal Mechanisms.

The observed behavioral and neuronal effects of dopamine manipulations follow basic assumptions of RL. Artificial dopamine excitations gradually increase lever pressing, cue approach, place preference, reward choice and dopamine response to the reward-predicting stimuli over repeated trials (Fig. 4 *A*, *B*, and *D*), indicating increasing reward value compatible with positive reinforcement. Artificial dopamine inhibitions increase choice of alternative places and options (Fig. 4*C*) that result in less dopamine inhibition compatible with negative reinforcement. Among other effects, such dopamine signals may explain risk attitude (*SI Appendix, SI Text 3*).

The time courses of artificial dopamine excitations effective for prediction updating correspond roughly to those of natural dopamine excitations evoked by natural RPEs in monkeys (35) and mice (22, 25) [stimulated population activity blurs the dopamine response heterogeneity (49)]. Thus, the artificial dopamine excitations seem to mimic positive dopamine RPE signals that are naturally elicited by better-than-predicted rewards, and artificial dopamine inhibitions may mimic negative dopamine RPE signals elicited by worse-than-predicted rewards. By corresponding to artificially evoked dopamine signals, natural positive and negative dopamine RPE signals may have similar reinforcing functions as the artificial signals.

### Who Updates Reward Predictions, RPE or Dopamine Signal?

While the optogenetic stimulation experiments demonstrate prediction updating by artificial dopamine signals, the source of the reinforcement effect is debatable: Is it the RPE itself or the dopamine signal? In the laboratory, artificial dopamine signals are effective in prediction updating, without natural reward being involved. Outside the laboratory, only natural rewards that are better or worse-than-predicted can elicit such dopamine RPE signals. Such natural RPEs elicit RPE signals in most dopamine neurons, and in subpopulations of non-dopamine neurons (63–71), that would be suitable for updating reward predictions. A similar mechanism may work with dopamine surges induced by drugs of abuse, as the surges mimic chemical effects of positive dopamine RPE signals. In this way, the dopamine neuron might constitute an implementation of Klopf’s Hedonistic Neuron that maximizes pleasure and minimizes pain (18). After all, outside events can act on behavior only via the brain, and it is the brain’s activity that mediates the behavior.

The updating effects of dopamine RPE signals extend the recursion of RL to neuronal responses: The dopamine RPE signal updates the prediction, as suggested by the artificial stimulation experiments (31, 35). The prediction determines the next dopamine RPE signal, which updates the next prediction that determines the following dopamine RPE signal, and so forth.

As a potential challenge to their behavioral effects, dopamine excitations might attract agents to unpredicted small risky rewards eliciting positive RPEs rather than fully predicted larger safe rewards no longer eliciting positive RPEs beyond initial learning. While this might indeed happen for a while, predictions of the smaller risky rewards established by the RPEs via RL unlikely exceed the earlier established predictions for the larger safe rewards. Hence, the positive RPEs and dopamine excitations elicited by these predictions would scale with their reward value and overall attract the agent to the better safe rewards. Only when the risky rewards are valued subjectively higher than the safe rewards because of risk attitude would the animal be attracted to the gamble.

### Downstream Effects of Dopamine RPE Signal.

The behavioral effects of dopamine RPE signals likely involve neurons in postsynaptic structures. The effects of dopamine action potentials on striatal and cortical neurons depend on axonal branching, heterosynaptic- and autoreceptor-controlled dopamine release, dopamine receptor type, dopamine reuptake transporter activity, and local plasticity. In dopamine terminal areas, the heterogeneity of striatal and cortical neuron activity adds further specificity. In this way, dopamine activity affects functions of the striatum, rest of basal ganglia, frontal cortex and ultimately the motor system that execute the approach and withdrawal behavior attracted by dopamine excitations and inhibitions. For details on postsynaptic effects of dopamine signals, see *SI Appendix, SI Text 4*.

Computer implementations of TD RL demonstrate that dopamine-like RPE signals are useful for learning Backgammon, Atari, Go, Chess and Shogi games, with added deep neural networks for efficiency (72–74). Neurobiological experiments suggest that dopamine neurons are involved in learning. Optogenetic dopamine excitations mimicking dopamine RPE signals are effective for learning behavioral and neuronal reward predictions (25, 27, 31, 35). Dopamine application induces synaptic plasticity in the striatum and frontal cortex (75–77). N-methyl-D-aspartate (NMDA) receptor knockouts in mouse dopamine neurons result in reduced dopamine burst firing and deficient acquisition of conditioned place preference, operant responding and T-maze choice, without impairing other learning functions, spatial memory and novelty recognition (78, 79). Dopamine D1 and D2 receptor blockade and knock-down in monkey frontal cortex and striatum impair spatial-delayed stimulus and visual discrimination learning (80, 81). Blockade of dopamine receptors impairs striatal plasticity (82, 83). Thus, dopamine signals seem to be necessary and sufficient for simple forms of prediction and reward learning.

### Conclusions.

The artificial manipulations suggest that dopamine RPE signals provide positive and negative reinforcement and attract behavior toward better rewards and away from worse rewards. As likely underlying neuronal mechanism, dopamine RPE signals update behavioral and neuronal reward predictions that determine the next RPE and dopamine RPE signal in the recursive manner of RL.

## Dopamine Mechanism for Reward Maximization

Basic RL formalisms describe how RPEs update reward predictions, and TD RL extends these descriptions to predictions that serve as higher-order rewards. Economic choice theory defines subjective reward value as the crucial variable to be maximized. Within these frameworks, the proposed reward maximizing mechanism relies on the empirically demonstrated RL function of the dopamine RPE signal.

### Foundation in RL Formalism.

RL engages a recursive mechanism between rewards, RPEs and predictions: A reward that is better-than-predicted elicits an RPE that updates the prediction that determines the RPE elicited by the next reward that updates the prediction again that determines the next RPE, and so forth. This mechanism kicks in when agents improve themselves by finding rewards that are better-than-predicted from past experience and elicit positive RPEs.

**Fig. 5.**
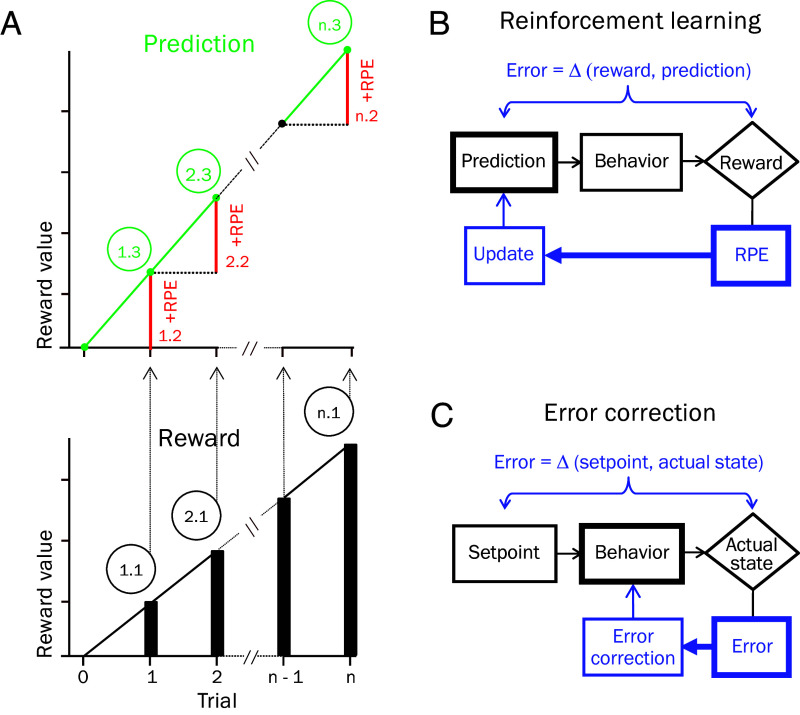
Reward maximization via recursive RL. (*A*) Cycles of RL increase reward. A better-than-predicted reward (step 1.1) elicits a positive RPE (red; step 1.2) that increases the prediction (green; step 1.3). As RPE equals reward minus prediction, only a reward exceeding the increased prediction (step 2.1) can elicit a similar positive RPE again (step 2.2), which increases the prediction further (step 2.3). The cycles of reward, RPE, and prediction continue until no further better reward can be obtained (steps n.1 to n.3). (*B*) Reward prediction updating by RPE. Being attracted to RPEs, predictions can grow and shrink without predetermined bounds. (*C*) Error correction with steady-state setpoint. An error brings the actual state back toward the setpoint but, in contrast to RL, does not affect the setpoint. Thus, the actual state can only vary around a setpoint.

The proposed recursive reward-maximizing mechanism advances in cycles (Fig. 5*A*). A cycle starts when an agent’s action results in a better reward than predicted (step 1.1). The better reward elicits a positive RPE (step 1.2) that increases the prediction via RL (step 1.3). Given that RPE equals reward minus prediction, the same reward after an increased prediction elicits a smaller positive RPE (Fig. 1 *B* and *C*), and a similar positive RPE as before requires a reward that exceeds that increased prediction.

When an action results in that better reward, the next cycle starts (step 2.1); the better reward elicits a positive RPE (step 2.2) that increases the prediction further (step 2.3). The cycles repeat themselves. In each cycle n, a better-than-predicted reward (step n.1) results in a positive RPE (step n.2) that increases the prediction further (step n.3). The iteration stops, and the maximal reward is reached, when the agent’s actions fail to obtain further better rewards that elicit positive RPEs. The result of this reward-RPE-prediction cycle is an upward spiraling iteration toward the best possible reward agents can obtain.

Thus, the crucial point for the maximization mechanism is the repeated occurrence of substantial positive RPEs. As the prediction grows with each positive RPE, the reward also needs to grow to keep exceeding that growing prediction and elicit a positive RPE (Fig. 5*A*). By contrast, repetition of reward itself only drives the prediction to asymptote without engaging a reward-maximizing cycle (Fig. 1*C*).

The RPE-induced mechanism protects also against losses. Encountering a lower reward than predicted elicits a negative RPE, which lowers the prediction. The lower prediction allows an agent to more readily find a reward that can exceed the low prediction and elicit a positive RPE, thus entering the reward-increasing cycle and recovering the loss.

In contrast to RL, simple error correction mechanisms do not support similar reward maximization. Although the effects of RPEs on predictions reduce the error between reward and prediction, standard RL is not a steady-state error correction mechanism that works around a fixed setpoint. RL is essentially unconstrained and able to increase predictions without preset bounds (Fig. 5*B*). By contrast, the error in steady-state error correction acts directly on the behavior and brings the system back to its setpoint (Fig. 5*C*), which applies also in homeostatic RL with a fixed allostatic setpoint (84). Hence, an error correction mechanism that brings the error down would not serve the proposed reward maximizing mechanism that requires a maintained substantial RPE and the essentially unbounded reward prediction of RL.

### Reward Maximization via Dopamine Signals.

The recursive characteristics of RL explain how RPEs can iteratively increase rewards. The involvement of dopamine RPE signals derives from their self-stimulation effects, from their updating of reward predictions, and from their coding of subjective value that defines reward benefits for biological organisms.

**Fig. 6.**
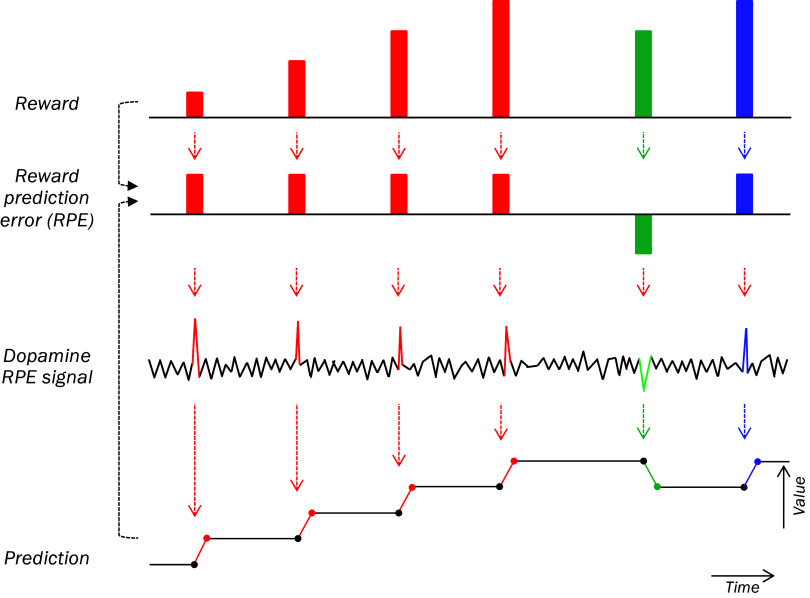
Reward maximization via dopamine RPE signals. Red: Unpredicted rewards received by an agent elicit positive RPEs and positive dopamine RPE signals that increase reward predictions by sensory cues via RL in Pavlovian and operant procedures. As RPE equals reward minus prediction, an unchanged reward after an increased prediction elicits a smaller or no positive RPE and dopamine RPE signal; only better rewards than before can elicit similar positive dopamine RPE signals again. Agents attracted to positive dopamine RPE signals need to find increasingly better rewards to continue receiving such RPE signals (*Top*). Green: Worse-than-predicted rewards elicit negative RPEs and negative dopamine RPE signals that decrease reward prediction via RL, and smaller rewards than before can elicit positive RPEs and positive dopamine RPE signals (blue). Thus, the key to the proposed reward maximization mechanism is the attraction to positive dopamine RPE signals.

The dopamine reward maximization process advances in analogy to the behavioral reward maximization process (Fig. 6, red). The crucial variable is the dopamine RPE signal that agents try to acquire or avoid, as shown by the self-stimulation experiments. To obtain a positive dopamine RPE signal, an agent needs to find and choose an action that results in a better reward than predicted. Once obtained, the positive dopamine RPE signal updates predictive neuronal signals of decision variables via RL, such as object value and action value that constitute inputs to competitive decision processes (6–8). Given that RPE equals reward minus prediction, the now increased prediction requires a better reward for eliciting a similar dopamine RPE signal as before, whereas a similar reward again would only elicit a smaller or no RPE and dopamine signal. When the agent has acquired that better reward, it would again elicit a positive dopamine RPE signal. That signal updates predictive object value and action value signals for the next decision again via RL.

The RL recursion cycles: Every time the action of the agent results in a better reward than predicted, the elicited positive dopamine RPE signal increases the prediction, and an even better reward is required to elicit a similar dopamine RPE signal. The process only stops when further actions fail to result in better rewards than predicted capable of eliciting positive dopamine RPE signals.

The mechanism deals also with worse rewards than predicted that elicit negative dopamine RPE signals, but the details differ slightly. When an action results in a worse reward than predicted (Fig. 6, green), that reward elicits a negative dopamine RPE signal, which decreases the reward prediction according to RL. Against that lower prediction, the next reward can be worse than before to elicit a positive RPE and dopamine excitation and is thus easier to obtain. That elicited positive RPE increases the prediction back toward where it had been before (Fig. 6, blue). The iteration toward better rewards can now resume from this lower reward demand to remedy the loss.

According to TD RL concepts, all events predicting the ultimate reward constitute higher-order rewards, and dopamine responses to ultimate and to higher-order rewards both reflect RPEs. In the case of TD RL, the mechanism would maximize overall state value rather than individual rewards (that might be lower when advancing through them to higher state value).

As dopamine neurons code temporal RPEs (first derivative of reward value over time), even a reward with a fully predicted amount elicits a dopamine excitation when that reward occurs at an unpredicted moment in time (50). Thus, dopamine excitations do not only occur with rewards that are better-than-predicted but also when rewards with well-predicted subjective value occur at unpredicted moments. In analogy, dopamine neurons are inhibited at the moment at which an omitted reward would have occurred, even when the reward with its fully predicted amount occurs later. This temporal sensitivity extends the situations in which the dopamine RPE signal may occur and can result in the proposed reward maximization mechanism.

### Conclusions.

The proposed reward maximization mechanism relies on the causal function of the dopamine RPE signal within the recursive RL mechanism. The crucial factor is the experimentally demonstrated attraction to positive RPEs; as such RPEs increase predictions, agents can only obtain such positive RPEs again by searching for increasingly better rewards that exceed the recursively increasing predictions. Negative dopamine RPE signals lower the predictions that can be more easily exceeded by available rewards, which facilitates the attraction to positive RPE signals. Thus, the dopamine RPE signal attracts agents to better rewards and away from worse rewards. The mechanism iterates toward maximal reward until no further better rewards can be obtained.

## Extensions and Outlook

Obtaining better rewards and avoiding worse rewards are crucial for survival and evolutionary fitness. Dopamine RPE signals embedded in recursive RL support these tendencies and may result in reward maximization. Although humans have never been tested for dopamine self-stimulation behavior, they show dopamine RPE responses that correspond to those in monkeys and rodents (85–87). Thus, the proposed reward maximization mechanism may apply also to humans with their more sophisticated and verbally expressed behavioral tendencies. The proposed mechanism may well constitute a blueprint for reward maximization that could extend beyond dopamine neurons and even work without explicit RPE signals in individual neurons.

### Role of Other Neuronal Reward Signals.

Given its biological importance, reward maximization may involve also other brain systems and mechanisms. Besides dopamine neurons, electrical self-stimulation compatible with RL is effective in the cerebral cortex, subcortical structures, and cerebellum (20, 88), where selected neurons code RPEs (63–71) that may update reward predictions and participate in reward maximization via RL. Distinct from reward maximization embedded in recursive RL, economic theories conceptualize reward maximization based more directly on choices (4, 5). Compatible with these concepts, neurons in the primate orbitofrontal cortex, striatum, and amygdala code economic decision variables such as object value, action value, and chosen value (6–8) and attract choices toward options associated with the highest reward-related neuronal excitation (9–11). Thus, there exist at least two distinct fundamental brain mechanisms for reward maximization, which is not surprising given its importance for individual survival and evolutionary selection.

The circumscript neuronal reward maximization account may also apply to everyday behaviors. While dopamine RPE signals are observed with high temporal specificity in restricted laboratory settings, satisfaction and happiness in real life often involve less explicit events and situations that may engage cortical and subcortical systems with their own mechanisms beyond prediction error coding. Then the proposed dopamine maximization mechanism might only be a template for mediating well-being by various motivational and cognitive neuronal systems.

Neuronal reward signals are abundant in the orbitofrontal cortex, striatum, and amygdala (6). Animals may find these signals attractive when they relate to particularly good rewards, irrespective of eliciting RPEs. I will always go for a good piece of chocolate or a nice serving of guacamole, even if it is not better-than-predicted, simply because I like them so much. Thus, neuronal signals for rewards not eliciting RPEs may be strong motivating factors for maximizing reward irrespective of dopamine excitations. In any case, the attraction of neuronal signals for RPEs and error-free rewards seems to be related to reward processing: While neurons in the visual cortex are excited by visual stimuli, animals are not known to come back for more visual cortex stimulation.

A dopamine involvement in the attraction to better reward may be a feature of efficient brain design (89). The attraction may be linked to allostasis in which the regulatory setpoint changes for optimal functioning (and maximal reward) (90). The flexible setpoint is equivalent to the reward prediction of RL, is updated by the current reward, and serves as reference for the next reward. Thus, a system using RPEs requires progressively stronger stimuli to achieve the same sense of well-being (89). The well-being does not derive from absolute reward value but from better reward relative to the current state. The proposed dopamine reward maximization mechanism may provide a neuronal basis for such a principle.

The benefits of reward maximization for survival and evolutionary fitness become particularly evident when resources are scarce. Maximization mechanisms likely exist in some form in all animals that have emerged from millions of years of evolutionary selection. As dopamine neurons exist in a wide range of these species, some form of dopamine mechanism of reward maximization may be implemented in the widely varying cognitive capacities of species, including habits and goal-directed behavior (91), and involving model-based and model-free RL (92). Interestingly, while complying with simple Pavlovian model-free RL mechanisms, dopamine RPE signals incorporate also model-based processes when tested with reward probability distributions and stimulus-reward reversals (39, 93).

### Evolutionary Benefits, at a Cost.

The desire for increasing rather than static rewards is not new. Goethe reportedly stated "Nothing is harder to bear than a succession of fair days". Without obviously being aware of dopamine mechanisms, Goethe called for stimulation from better-than-predicted days. A similar mechanism may underlie the happiness-income paradox (94); after a short period of higher income, the happiness dissipates and more income is required for maintaining the happiness. The hedonic treadmill provides a further example (95); after having obtained a desired reward, agents find less satisfaction in the same reward predicted by the achieved level and are stimulated by the prospect to obtain better rewards, which in many ways constitutes a beneficial mechanism. A downside is drug consumption when agents chase increasingly larger doses of harder drugs to maintain satisfaction.

Despite its obvious benefits, the quest for ever-better rewards has a price: We are rarely satisfied with what we have, and we become restless and search for ever-better rewards because their elicited dopamine excitation reinforces these behaviors. The resulting greed and overconsumption stifles initiative and creativity and absorbs resources and energy that endanger the survival of civilizations (96, 97). Therefore, it may be useful to stop the vicious cycle, renounce the "ever more" principle, propose stabilization, and reset run-away references. Stabilization would require enormous energy to counteract the built-in maximization mechanism, and outright resets would require painful losses. Economic decline, natural disasters, and wars might inadvertently provide such resets. Despite their tragic nature, they would restart the cycle and hopefully make agents more modest and wiser in the process and give them a chance to find better ways to deal with it the next time around. Thus, neuronal reward maximization may be beneficial for immediate survival and evolutionary fitness, but it may also strain our well-being by pushing us to the edge.

## Supplementary Material

Appendix 01 (PDF)

## Data Availability

All study data are included in the article and/or SI Appendix.
