# Supplementary material for: A dopamine mechanism for reward maximization
Source: Proc Natl Acad Sci U S A. 2024 May 8;121(20):e2316658121. doi: 10.1073/pnas.2316658121 (PMC11098095; doi:10.1073/pnas.2316658121)
Supplement: Supplementary file 1 — Appendix 01 (PDF) [file pnas.2316658121.sapp.pdf]

PNAS

Supporting Information for
A Dopamine Mechanism for Reward Maximization

Wolfram Schultz
Email: ws42@pm.me

This PDF file includes:

Supporting Information Text 1 - 4
Figures S1, S2
SI References

SI Text 1: Diversity of dopamine signals

Recent studies on mammalian dopamine neurons report a variety of neurophysiological responses that seem confusing and require a closer look (1, 2). We may be used to the notion that each brain system has one and only one function. The primary visual cortex seems to process visual information and nothing else, and the motor cortex, prefrontal cortex, cerebellum, basal ganglia and amygdala may do so likewise for their respective functions. Thus, the variety of dopamine signals that has been known for some time (3) seems incompatible with the notion of 'one brain system equals one function'. From the dopamine reward prediction error (RPE) response we know that dopamine signals are involved in reward processing, and from the deficits in Parkinson's disease we know that dopamine neurotransmission is involved in movement, but how can we understand the other dopamine functions?

Overview. The dopamine RPE response is not the only phasic signal of dopamine neurons (1-6). The RPE response is preceded by a short, unspecific, prediction-dependent excitation elicited by salient events, including punishers, stimuli resembling rewards, and novel stimuli (7). After the initial subsecond salience and RPE responses, dopamine neurons show slower and heterogeneous excitations and inhibitions with a large range of sensory and motor events that generate behavioral activation (8-12). Many of these dopamine activities in rodents may reflect substantial, task-unrelated limb movements that generate widespread cortical activity in mice during small mouth and limb movements (13) and in monkeys during spontaneous movements (14). By contrast, small, precise eye and limb movements fail to elicit such slower dopamine changes when controlling for unrelated movements in monkeys (15-17).

Multiple RPE signal components. Rewards and reward-predicting stimuli elicit a sequence of processes that reflect their detection, identification and evaluation. The phasic dopamine signal, similar to responses in other neuronal systems, has at least two components that correspond to these sequential processes (18-21). Both components depend on prediction. The initial component response occurs with a large range of environmental events and depends on the attention elicited by an event (Fig. 2 brown); the response decreases with the decreasing attention elicited by an increasingly predicted event (dotted brown line). The main component constitutes the bidirectional dopamine reward prediction error (RPE) signal (Fig. 2 red); its positive response (neuronal excitation) reflects larger-than-expected reward (positive RPE), and its negative response (neuronal inhibition) reflects smaller-than-expected reward (negative RPE) (solid vs. dotted red lines; see also Fig. 1*A, B*). This component reflects the temporal difference (TD) of reward value (dr/dt) or, more specifically, codes the TD of reward utility (du/dt) (see also Fig. *3D, E*). Importantly, both components occur at reward-predicting stimuli and at ultimate rewards in compliance with basic notions of TD learning theory (22).

The initial-component dopamine response (Figs. 2 brown, S1*A*) occurs with novel, neutral, rewarding and aversive stimuli, where it increases with physical impact, novelty, surprise, similarity to reward (generalization), and rewarding context (17, 19, 23-25). The response most likely reflects the salience common to these events. Saliency may also explain the dopamine response decay with decreasing novelty during learning (26, 27), underlie the dopamine response to reward identity shifts (28), reflect the prediction-induced response reduction to punishers (29), and explain the somewhat slower value-independent dopamine release (30). The initial-component response transfers during learning from primary visual events or aversive shocks to stimuli predicting

these events (24, 29), thus reflecting the unpredictability of the eliciting event (Fig. 1*A*) but without showing inhibition with event omission like the negative dopamine RPE response (Fig. S1*B*) (24), which suggests salience rather than bidirectional prediction error coding. Thus, the initial component may constitute a default signal that reports novel or poorly identified environmental events that are potential rewards and should be approached to not miss a reward; therefore, such a neuronal signal would be beneficial for individual animals and evolutionary selection (21).

The distinction between the initial component and the main RPE signal requires temporal resolution in the ten-millisecond range; accordingly, some modern cellular imaging techniques with slower time courses, despite their elegance, may not allow distinction between the initial, prediction-dependent component and the genuine bidirectional RPE signal. The distinction would be helped by independent variation of the sensory and reward parameters of the eliciting event and multivariate data analysis (19).

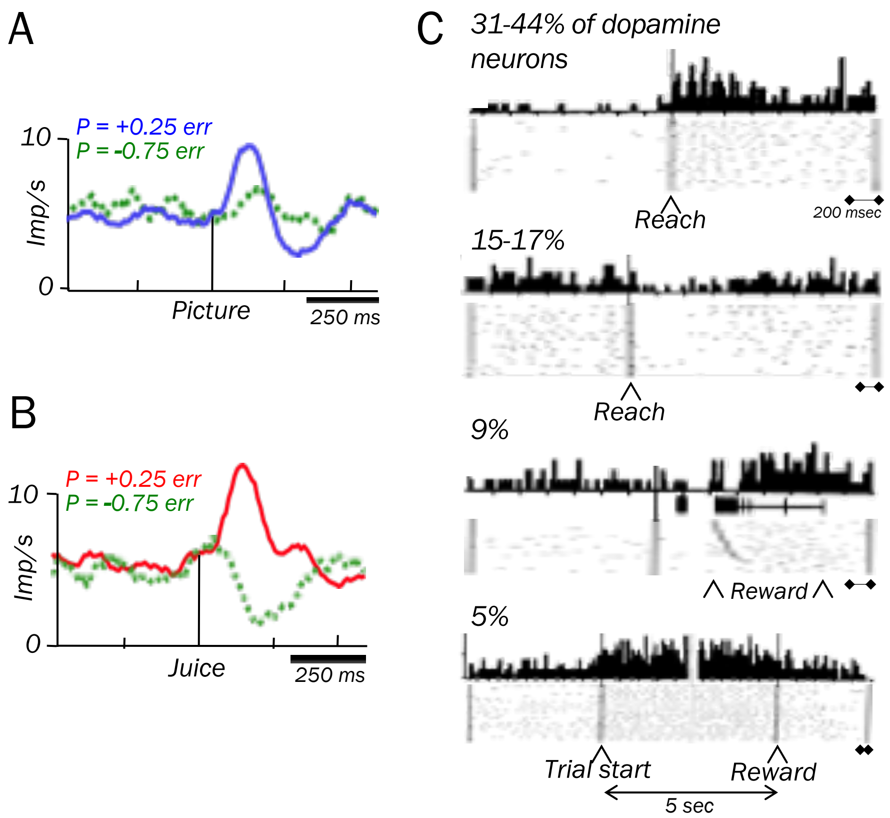

Fig. S1. Diverse dopamine responses in addition to reward prediction error (RPE) signal. (*A*) Prediction-dependent unidirectional attentional coding of non-rewarding pictures in monkey: excitation by surprising appearance of probabilistic picture (P = 0.25), but absence of inhibition by picture omission (P = 0.75) (population average from 14 dopamine neurons). Reproduced from own work (CC-BY) (24). **(*B*)** For comparison: bidirectional juice RPE coding in same dopamine population as *A* (CC-BY) (24). **(*C*)** Heterogeneous slow phasic excitations and inhibitions in different monkey dopamine neurons during behavior. Time marker applies to all subpanels (200 msec). Reproduced from own work (4, 8).

Aversive responses. Many studies, including our own, report excitations by aversive stimuli in some dopamine neurons (19, 23, 32). In analogy to rewards, punishers elicit sequential components of detection, identification and evaluation. Dissociations of these components suggest that most reported dopamine excitations by aversive stimuli reflect the sensory impact of the first saliency component rather than genuine aversive negative value of the punisher (19, 23). Most dopamine neurons either do not code aversiveness at all (19) or show inhibitions that reflect the negative reward value (second component) (19, 29, 31). The inhibitory punisher responses are compatible with full value coding from positive (excitation with reward) to negative (inhibition with punishment) and thus can be aligned with the RPE account of dopamine signals. Further, dopamine neurons are excited by omission of punishment, which constitutes a negative prediction error of negative value and thus a positive RPE (25, 29). Also, as relief from punishment is rewarding, its prediction elicits a dopamine excitation (28, 31). In contrast to these responses that are compatible with the standard RPE account, a population of outlying dopamine neurons projecting to the posterior striatum shows excitation by aversive air puff but not by other

aversive stimuli; as these neurons are not excited by punishment omission, they code neither general reward value nor value prediction error (31). Thus, apart from these exceptions, the 'canonical' dopamine RPE signal reflects reward value, together with its early sensory detection, rather than punishment.

Multiple phasic dopamine functions. In addition to the composite RPE signal, dopamine neurons show distinct slower and lower heterogeneous excitations and inhibitions that last from hundreds of milliseconds to several seconds and minutes; they arise in a wide variety of well-established tasks (1-3) (Fig. 2 blue).

These changes were first observed in monkeys. They occur in various task epochs during or before large reaching movements, during consumption of expected rewards or over whole trial durations (Fig. S1*C*) (4, 5, 8). The movement changes are not precisely related to movement onset, fail to vary with postural adjustments and small reaching movements, and do not occur with precise and well-controlled elbow, hand and eye movements (15-17, 34, 35). Further, some dopamine neurons show ramping activity in anticipation of risky rewards (36). In rodents, which may show substantial task-unrelated behaviors (13), dopamine neurons show even larger varieties of slow and heterogeneous excitations or inhibitions that vary with movement speed and velocity (12, 37-42) and may also reflect inadvertent visual, auditory and proprioceptive sensory stimulation during on-going behaviors. Some of these activities ramp up over several seconds toward the reward and last beyond, reflecting sensory stimulation, movement, reward expectation, motivation and general behavioral activation (10, 43-47); the ramp is often absent without sensory feedback (27, 48-54). These changes are independent of any prediction, including reward prediction, and thus do not seem to reflect RPEs.

While these activities are slower and lower than the phasic RPE signal with rewards and reward-predicting stimuli, they might contain small RPE responses. TD implementations often assume small RPEs that backpropagate during learning via imaginary intervening stimuli from primary reward to the next preceding stimulus (55-57). However, biological dopamine neurons do not respond to imagined stimuli (58); accordingly, monkey dopamine neurons do not show ramps in tasks with short stimulus-reward intervals and devoid of intervening events or movements (55), except with risky rewards as noted above (36). Interestingly, ramps are not essential for biologically plausible TD implementations when using larger backward steps (59). In rodents, dopamine neurons show ramps during learning (60). Ramps occur also during well-established tasks with intervening stimuli that may generate small RPEs arising from inaccurately perceived temporal reward predictions ('state uncertainty') (61). Dopamine neurons in monkeys tested in such richer tasks with multiple stimuli may also show ramping activity reflecting small RPE responses.

The large variety of sensory, motor and rewarding events eliciting slower dopamine changes in monkeys and rodents may not only reflect specific behavioral processes but also include underlying general processes such as motivation, arousal and behavioral activation. The more stimuli and movements engage the animal, the more frequent and stronger seem to be the slower dopamine changes. These dopamine changes are often difficult to distinguish from the more phasic RPE signal and are sometimes lumped together into a single phasic dopamine change, in particular in rodents that often show multiple parallel behaviors with inadvertent, task-unrelated limb movements and locomotor activity (13). Recording methods should have temporal resolution of tens of milliseconds, and statistics for the neuronal analysis should use multivariate methods (12). Appropriate regression models should account for potential intercorrelations between reward expectation, movement speed, vigor, motivation, arousal and behavioral activation.

Besides these slower phasic dopamine changes, tonic levels of extracellular dopamine in the striatum may have permissive influences rather than phasically driving motor and cognitive functions, as the efficacy of dopamine receptor stimulating drugs in alleviating Parkinsonian deficits indicates (Fig. 2 light blue, ‘tonic’). Modulation of the influence of such tonic dopamine 'fuel for the brain' may also underly the behavioral effects of dopamine agonists and neuroleptics.

Absence of dopamine responses despite reward prediction or delivery. Dopamine neurons fail to respond to stimuli and rewards that are fully predicted (8); when in addition general behavioral activation is limited, dopamine neurons may not show any task modulation at all (15). The first stimulus in such tasks would predict the ultimate reward, and its appearance at an unpredicted time should elicit an RPE response. However, due to the delay to the reward, temporal discounting (62) may reduce this response to a level that is indistinguishable from neuronal noise.

Multiple neuronal functions in other brain systems. The possibility of individual brain systems having more than one function is not entirely new. Multiple functions are classically seen in phylogenetically older brain structures like the amygdala whose neurons are not only involved in fear conditioning but also process reward information (63) and exploratory states (64). The same amygdala neurons code reward value early in a trial and reward choice at trial end (65). In the hippocampus, place cells change their focus depending on locally available reward (66). Even in the cerebellum with its staunchly motor function, optogenetic stimulation drives behavior

toward reward (67). In the monkey prefrontal cortex, the same neurons process several task components (68). Even neurons in primary visual cortex code reward information (69) and specific aspects of face motion together with arousal states (70). Neurons in primary motor cortex code also visual and, classically, somatosensory information (71, 72). These mixed selectivities might constitute examples for energy-saving in the brain by using the same neurons for different processes. Hence, the notion of one brain system having just one function might be an unrealistic myth, and dopamine neurons are no exception.

Summary. Phasic dopamine signals have multiple functions. The RPE signal is composed of an initial component that reflects the salience elicited by the detection of any sufficiently strong event, including reward-predicting stimuli and rewards (Fig. 2 brown), and a main component that reflects the true bidirectional RPE (red). Somewhat slower, non-RPE dopamine changes lasting for seconds or minutes (blue) occur during movement initiation or reward delivery, or reflect the TD ramp, reward risk, reward expectation, sensory stimulation or movement, or common underlying processes of general arousal or behavioral activation. An even slower change or outright tonic level of striatal and cortical dopamine concentration (light blue) is required for well-functioning motor and cognitive processes, as the deficits in Parkinson's disease and the behavioral effects of dopamine agonists and neuroleptics attest. Thus, the recognition of the slower dopamine signals provides for a more complete appreciation of dopamine functions. However, as interesting as they are, the slower dopamine functions do not play a major role in the proposed dopamine reward maximization mechanism, which relies on the RPE signal.

SI Text 2: Economic choice and utility

Economic utility is a mathematical representation of preferences (73) and thus provides a metric of subjective reward value. Rather than estimating subjective value on an ad-hoc basis, definitions of utility functions convey the advantage of mathematical functions in predicting events that have not been used for their estimation. In economics, this prediction concerns the choice of goods, also called rewards in biology. Utility is not objectively measurable but can be inferred from observable choice. Rewards are typically uncertain and risky, which contributes to the subjectivity of reward value. Thus, utility functions can be estimated from choice under risk. Choice under risk is also the basis of the four utility axioms of Expected Utility Theory (EUT) that define utility maximization under risk and provide a conceptual foundation for estimating utility from choice (74-76).

Definition of risk. Risk has three basic connotations, variability, loss, and gain. Among the many definitions of risk, economic risk concerns the higher statistical moments of outcome probability distributions, notably variance (second moment, a measure of spread), skewness (third moment, a measure of asymmetry), and kurtosis (fourth moment, a measure of symmetric outliers). This definition of risk requires fully known probability distributions (whereas the uncertainty in incompletely known probability distributions is referred to as ambiguity). Thus, economic risk focusses on variability and considers any outcome below the mean of a gamble as (relative) loss and any outcome above the mean as (relative) gain. A different, popular view defines risk as the probability of loss, which equals negative probabilistic value. This view focusses on loss and considers probability to represent variability, but the definition does not include the potential gain (without which risk-taking would be useless). The intuitive, everyday notion of risk often comprises a weighted combination of both definitions. Here I will only follow the economic definition.

Basic choice design. In the most simple and controllable version, a monkey chooses on each trial between two discrete and distinct options (binary choice) that appear simultaneously at the right and left of the center on a computer monitor in front of the animal. The animal chooses its momentarily preferred option by eye movement or joystick movement, or by contacting a specific spot on a touch-sensitive computer monitor. After the choice, the chosen option, and no other option, is paid out to the animal. Thus, the option set is collectively exhaustive (all options are included, and every option can be chosen) and mutually exclusive (only one option can be chosen in a trial).

The definition of economic risk based on reward probability distributions encourages simple experimental designs that demonstrate meaningful choices in rhesus monkeys. A most simple design comprises a 'safe' option with a specific reward amount and a 'risky' option with several probabilistically alternating reward amounts (whose probabilities sum up to $P = 1.0$). Each option is represented by a distinct quantitative stimulus composed of one horizonal bar (safe option) or several bars (risky option), each inside a vertical rectangle (Fig. S2*A* top). The vertical position of each bar inside the vertical rectangle indicates the reward amount the animal would receive with a specific probability if it chooses that option. For example, with equiprobable choice options, two bars indicate two different reward amounts, each of them occurring on half the trials ($P = 0.5$ each amount); the animal receives only one of these reward amounts when it chooses that option. Thus, the reward amounts of a

multi-amount option are collectively exhaustive (all amounts are included, and every amount can be delivered) and mutually exclusive (only one amount is delivered on a given trial).

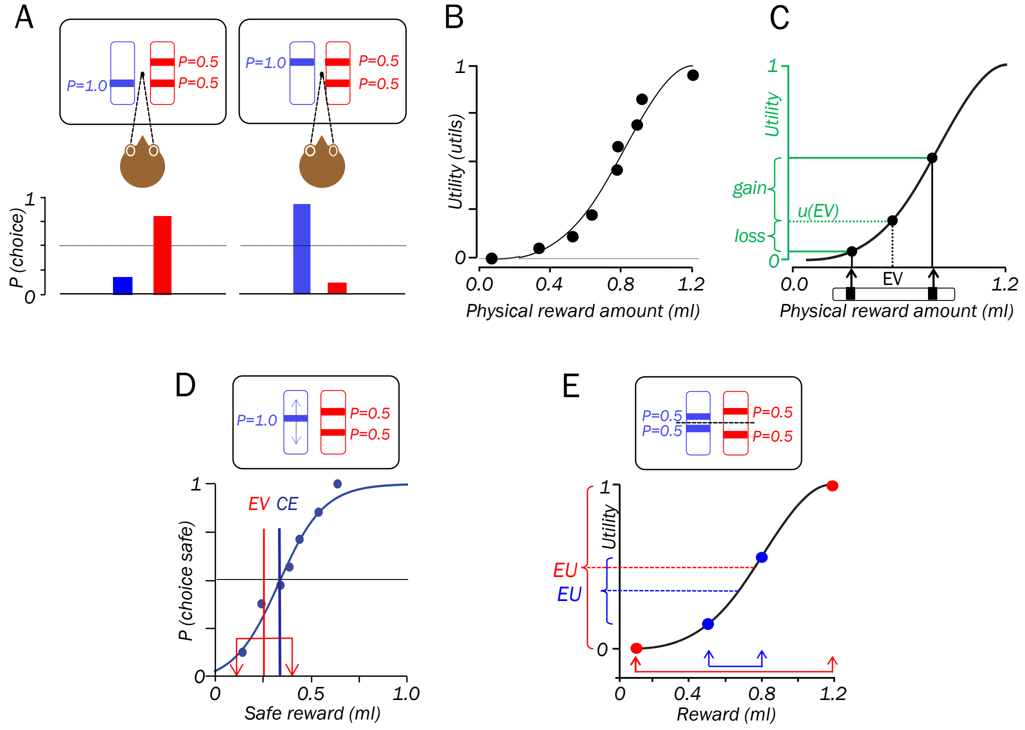

Fig. S2. Utility estimation and maximization during risky choice in monkeys**. (*A*)** Meaningful choice according to reward goodness shown by compliance with first-order stochastic dominance (FOST). Top: ocular stochastic choice between a safe reward (blue) and a risky reward (red, probability *P* (each amount) = 0.5). Left option set: gamble is always equal to or better than safe reward (gamble dominates safe reward). Right option set: safe reward is always equal to or better than gamble. Bar height within each rectangle indicates juice amount (higher is more). Both stimuli of each option set are presented simultaneously on a computer monitor in front of the animal. Bottom: choice probability of each option indicates compliance with FOST. The occasional choice of the non-dominating option reflects the nature of the stochastic choice process. **(*B*)** Utility function estimated from fractile chaining procedure. Higher variance defines more economic risk (red). Higher choice probability for riskier option indicates risk-seeking. Physical reward amounts are ml of juice. **(*C*)** Schematic showing how convexity of utility function predicts risk-seeking with binary gamble. Respective distances to utility of mean physical gamble amounts (u(EV); EV, expected (mean) value) indicate that large rewards are subjectively overweighted and small rewards underweighted. Hence, the utility gain from obtaining the large reward relative to mean utility is larger than the utility loss from obtaining the small reward, consistent with risk-seeking. Rectangle with 2 solid inside bars indicates binary equiprobable gamble (*P* = 0.5 each reward amount). **(*D*)** Utility maximization at choice indifference. The options have same utility (expressed as Certainty Equivalent, CE) when chosen equally frequently (P (choice safe reward) = 0.5; choice indifference); here, the animal chooses the gamble with the lower physical EV in half the trials, indicating that its choice follows utility rather than physical reward amount (note that the animal ranks rewards correctly according to their goodness, as shown in a). Top: options presented to the animal for psychophysical estimation. The safe reward occurs with probability P = 1.0 (blue); the risky rewards occur in random alternation with P = 0.5 each (red). Risk is defined as reward difference between bottom and top outcome (variance; no skew). (***E***) Utility maximization with same mean physical reward amounts. Projection of the two tested gambles onto a globally convex utility function (red and blue dots) indicates higher Expected Utility (EU) of riskier gamble (red) compared to less risky gamble (blue) despite same mean physical amounts (dotted line in top display). Note that the higher utility (EU, red) in this test with the same monkey explains the risky choices in panel *A* by value-seeking rather than risk-seeking. Panels *A, B* and *C* newly created, and panels *D* and *E* modified (CC-BY), from own work (77).

Meaningful risky choice. Any assessment of utility maximization rests on the assumption that animals' choices reflect the rank-ordered goodness of rewards; they should prefer unambiguously better rewards to worse rewards. This requirement can be tested formally by invoking first-order stochastic dominance in stochastic choice of probabilistic options, which is defined as every probabilistic option being at least as good as its alternative and better in at least one instance. Statewise dominance, as its most simple version, is defined by every single reward of the better (dominant) option being at least as good as the corresponding reward in the alternative (dominated) option and better in at least one instance. In a test of statewise dominance, a monkey chooses between a safe reward (Fig. S2*A* blue) and a risky option containing two equiprobable but mutually

exclusive rewards (*P* = 0.5 each) (red). Choice of the safe option results in delivery of a small reward on every trial, whereas choice of the risky option delivers either the same small reward or a larger reward (Fig. S2*A* left). Thus, the option with the occasionally (P = 0.5) larger reward is always objectively and subjectively equal to or better than its alternative, and never worse (given a positive value function, for example when satiety is ruled out); the risky option dominates the safe option. Monkeys choose the better (dominating) option on most trials (Fig. S2*A* bottom) (77) (occasional selection of the non-dominating option is typical for stochastic choice and may indicate exploration). Alternatively, preference for the risky option might reflect risk-seeking. To differentiate between the value-seeking and risk-seeking interpretations, the animal is presented with a safe reward that has the same amount as the top risky reward (Fig. S2*A* right): that safe reward (blue) is now always equal to or better than any reward of the risky gamble (red) and thus is the dominating option. Monkeys choose mostly the safe over the risky option (bottom) and thus follow the better reward. This choice pattern is not specific for two-reward gambles but applies also to three-reward gambles in which each reward occurs with *P* = 1/3 (78). The animals' choices also follow first-order stochastic dominance when both options have two probabilistic rewards (Fig. 3*A*). Thus, the animals' choices satisfy first-order stochastic dominance; they seem to understand their stimuli, they distinguish between good and less good options, and they choose the better option. Thus, the design can be used for estimating utility from choices and then for assessing utility maximization.

Utility estimation. Utility functions can be reliably estimated in laboratory settings during choice under risk from a sequence of psychophysically estimated CEs at choice indifference (choice of each option with *P* = 0.5) between an adjustable safe reward and a pre-set equiprobable gamble, using the so-called fractile or chaining procedure (79, 80). Starting with a CE for an equiprobable gamble containing the minimal and maximal tested reward amounts (each delivered at *P* = 0.5), the procedure assesses new CEs with progressively finer resolution by iteratively setting gamble rewards to previously estimated CEs, thus obtaining more closely spaced CEs across the entire test range (for details, see (77)). Then the CEs are used to fit a full utility function using multiple splines or power, exponential, logarithmic or multi-parameter functions. The fitted utility functions with reward amounts used for monkeys are often S-shaped, gradually steepening with increasing reward amounts (convex to the origin), then becoming more linear and flattening gradually with larger rewards (concave) (Fig. S2*B*) (77, 81, 82). Such sigmoid utility functions have been conceptualized before for humans (83, 84). Utility functions estimated for safe rewards using the Random Utility Model (85, 86) show similar profiles (87), suggesting that the nonlinear transformation from objective physical reward amount to subjective utility applies to both risky and safe rewards. The convexity may partly reflect the small reward amounts required for testing the large number of trials typical for monkey experiments, although monkey utility functions can also be concave (88) (the 'well-behaved' concave human utility function typically concerns more substantial amounts).

The convex curvature of the utility function with small reward amounts corresponds to the risk attitude observed in binary choice between a risky gamble and a safe reward (Fig. S2*C*): relative to the utility of the mean gamble reward u(EV), the utility gain from receiving the top gamble reward exceeds the utility loss from receiving the bottom gamble reward. Thus, the gain exceeds the loss, and the animal would prefer the risky gamble to the safe reward when their EVs are the same; it would be risk-seeking, exactly as shown empirically (Fig. S2*D* red), and choice indifference requires the safe reward exceeding the EV (blue). By contrast, the concave utility curvature with larger rewards indicates a lower gain from the top gamble reward, and the animal would be risk-avoiding. Such risk attitudes are indeed observed in monkeys with two-reward gambles and three-reward gambles (77, 78), confirming that utility functions estimated under risk predict choice under risk. Further, the utility functions obtained from stochastic choices have cardinal numeric properties (defined as being immune to offset and gain change). Utility functions with these properties are mathematically appropriate for neuronal response functions that themselves have cardinal properties (which allow z-score-normalized responses to be meaningfully averaged across neurons, as often done in neurophysiology). Thus, according to the general function of mathematical functions, utility functions, rather than ad-hoc estimated subjective values, allow prediction of choices that have not been used for estimating the utility function.

Compliance with EUT utility axioms and Prospect Theory. The estimated utility functions are valid within the constraints of EUT (linearly weighted probability, fixed reference point, restriction to gains). Choices of monkeys satisfy the first three EUT axioms within well-defined option sets; (i) the choices are complete by revealing preferences for all options (77); (ii) the choices are transitive and thus consistent (if option A is preferred to option B, and option B is preferred to option C, then option A should be preferred to option C) (35); (iii) the choices follow the trade-off between probability and reward amount defined by the continuity axiom (89). In tests of the independence axiom (IA), which is the fourth and most demanding EUT axiom, monkeys show some outright preference reversals and many graded preferences changes. These IA non-compliances suggest that subjective value does not only include the usually nonlinear weighting of reward amount (utility) but also nonlinear probability weighting; such subjective value defined by a combination of weighted amount (utility) and

weighted probability corresponds well to monkeys' choices (90). Thus, monkeys choose more consistently than humans who violate the independence axiom often and severely (91, 92). The long experience of monkeys with the experiment and its reward distributions might partly explain their good performance (93).

The IA tests demonstrate the importance of nonlinear probability weighting, which constitutes one of the tenets of Prospect Theory (94). When probabilities are presented in random alternation, monkeys overweigh the influence of low probabilities on expected utility and underweigh the influence of high probabilities, which is represented by an inverted-S shape of the probability weighting function (95) and resembles the weighting of instructed probabilities in human choices. By contrast, block-wise probability changes result in a regular-S shape of the probability weighting function (81), which resembles the weighting of experienced probabilities in humans. Thus, reward probability weighting is more variable than weighting of reward amount in utility functions. The larger variability may reflect the complexity of probability perception that requires memory of event occurrence and is more delicate than the perception of reward amounts that are closer to detection by primary sensory receptors. Thus, both reward amount and reward probability as components of reward value are subjective.

Further, monkeys' choices of safe rewards are generally compatible with the reference dependency of Prospect Theory. The utility functions adapt to changing reward distributions over several months (96). Finally, Prospect Theory suggests a convex loss function that is steeper than the gain function. When considering the full test range of reward amounts, reception of the small reward of a gamble might constitute a loss relative to the mean gamble amount. The convexity of monkeys' utility functions in the lower amount range might then correspond to the convexity of the loss function of Prospect Theory; however, the function is less, and not more, steep at such low values.

These properties of monkeys' choices suggest that utility functions provide a fundamental, concept-based and mathematically defined approach for expressing reward value on a subjective scale. Together with probability weighting, utility functions incorporate key features of subjective reward value that can be inferred from economic choice. The successful behavioral characterization of utility encourages investigations of its underlying neuronal signals.

Empirical maximization of utility rather than physical amount. Utility maximization can be empirically tested when monkeys choose safe and risky rewards equally frequently ('choice indifference'). With small rewards, where the utility function is convex, monkeys typically prefer risky to safe rewards when the two options have the same EV (equal mean amounts), as described above (Fig. S2*D* red) (35, 97); the animals choose both options equally frequently (choice indifference) only when the safe reward exceeds the EV of the risky reward (certainty equivalent, CE, in Fig. S2*D* blue) (77). Despite these different reward amounts, the monkey chooses each reward equally frequently, suggesting that the two options have the same utility (as we infer utility from choice). Thus, the animal chooses the larger (safe) reward only on half the trials. On the other half trials, it chooses the on-average smaller (risky) reward, although it could have chosen the larger (safe) reward. The compliance with first-order stochastic dominance confirms that the animal understands the stimuli and distinguishes very well between reward amounts (Fig. S2*A*). Thus, the animal behaves 'as if' it chooses the smaller (risky) reward because of its utility, and surely not for its smaller physical amount, which suggests that utility matters more to the animal than reward amount. (Use of the term 'as if' is an attempt to bypass the circular argument that utility is inferred from choices, and that the animal chooses to maximize the utility that is inferred from choices.) The dominance of utility over physical amount occurs also with larger rewards where the utility function is concave. Here, choice indifference occurs when the risky reward is larger than the safe reward (77, 78): by choosing the smaller safe reward on half the trials, the animal foregoes the larger risky amount and thus chooses according to utility rather than physical reward amount. Thus, their choices under risk demonstrate that monkeys choose 'as if' they are maximizing utility rather than physical reward amount.

The tendency towards utility maximization is also present in choices between two gambles with the same mean reward amount but different variance (Fig. S2*E*). This 'mean-preserving spread' provides a minimal-confound test of risky choice (98, 99), as both options have the same EV (mean physical reward amount), same number of rewards (N = 2) that occur with same probability (P = 0.5, small nonlinear probability weight), and no skewness. Using a previously estimated utility function (Fig. S2*B*), the projection of the gambles onto the function suggests that the riskier gamble has higher expected utility than the less risky gamble (Fig. S2*E*) (77). If monkeys choose both gambles equally frequently, they would choose according to their same EV (same mean amount), but if they prefer the riskier to the less risky gamble despite same EV, they would choose according to utility (specifically expected utility, EU). Indeed, the animals prefer the riskier gamble 'as if' they are maximizing utility rather than physical amount (see Fig. 2*B*). Thus, monkeys maximize utility irrespective of different or same mean physical reward amounts (Fig. S2*D, E*).

SI Text 3: Risk attitude explained by dopamine excitation and inhibition

The results from artificial manipulations of dopamine signals suggest that animals choose more frequently events that result in dopamine excitation as compared to events resulting in dopamine inhibition (100-103). This notion is interesting in light of risk-seeking and risk-avoiding. The two reward outcomes of binary gambles elicit respectively positive or negative reward prediction errors (RPE) relative to the mean reward; the RPEs induce respective dopamine excitations and inhibitions. Thus, agents' risk attitude may depend on the relative strength of dopamine excitations and inhibitions elicited by the RPEs of gamble rewards. The relative strength of dopamine excitations and inhibitions depend on the curvature of the agent's utility function coded by dopamine neurons (77). A convex (gradually steepening) curvature reflects overweighting of large over small gamble rewards, resulting in risk-seeking (Fig. S2*C*), whereas a concave (gradually flattening) curvature reflects underweighting of large to small gamble rewards, resulting in risk-avoiding.

In the simple case of a binary equiprobable gamble, and a convex utility function, the dopamine excitation from the larger-than-mean gamble reward (eliciting a positive RPE) would have a stronger effect on an agent's behavior than the inhibition from the smaller-than-mean gamble reward (eliciting a negative RPE). Here, the agent would prefer the gamble to a safe reward (or to a less risky gamble); thus, the agent would be risk-seeking. But in the case of a concave utility function, the dopamine excitation from the larger-than-mean gamble reward would have a weaker effect than the inhibition from the smaller-than-mean gamble reward; the agent would be risk-avoiding.

The relative strength of dopamine excitation and inhibition would differentially update reward predictions. An overweight of the dopamine excitation from the large gamble reward (positive RPE signal) compared to the inhibition from the small gamble reward (negative RPE signal; convex utility function) would result, via RL, in stronger gamble-predicting dopamine excitation by a riskier gamble as compared to a less risky gamble, even when both options have equal mean reward amount (Fig. S2*E*, red vs. blue). As a result, the stronger dopamine excitation would drive an agent toward the riskier gamble. Risk-seeking can be particularly beneficial when resulting in particularly valuable rewards, namely outliers in positively skewed gambles (78). To the opposite, an underweight of the dopamine excitation from the large gamble reward compared to the inhibition from the small gamble reward (concave utility function) would result in weaker gamble-predicting dopamine excitation by the riskier as compared to the less risky gamble and result in risk avoidance. Thus, the dopamine excitation and inhibition induced by gamble rewards may provide a neuronal basis for behavioral risk attitudes.

SI Text 4: Postsynaptic effects of dopamine signals

Modeling work suggests that phasic dopamine RPE signals are suitable to serve as effective teaching signals for reinforcement learning (RL). A temporal difference (TD) model using the kind of dopamine-like RPE signals recorded during electrophysiological experiments can mediate the learning of a series of spatial tasks in a similar manner as monkeys do in the laboratory (59). Artificial computer models using TD RL, often together with deep neural networks, acquire champion-level and superhuman performance in Backgammon, Atari, Go, Chess and Shogi games (104-107). The obvious architectural differences between the artificial computer models and biological brains stress the importance of the TD RL algorithm over the particular implementation of the algorithm (108). Nevertheless, we are dealing with real brains and need to assess how the biological dopamine RPE signal may affect the neuronal mechanisms mediating TD RL.

The neurophysiological responses of dopamine neurons in the midbrain constitute the first step of processing RPEs. To result in RL, this signal is propagated along axons to postsynaptic structures that implement RL. The implementation involves several subsequent steps:

(1) Axons of dopamine neurons branch profusely in the ventral and dorsal striatum (nucleus accumbens, caudate nucleus, putamen), frontal cortex and amygdala (109). Axonal conductance can fail at any axonal branch and reduce action potential frequency there (110, 111). Such and other mechanisms may underlie the known heterogeneous dopamine release in postsynaptic structures (112). Thus, the dopamine action potentials may not propagate into the whole axonal arborization tree but may be selected depending on specific terminal regions.

(2) Once an action potential reaches a varicosity, its action on dopamine release is subject to local influences. Dopamine release is regulated by presynaptic dopamine autoreceptors (113, 114) and by rich presynaptic interactions with other neurotransmitters (115). For example, acetylcholine modulates striatal dopamine release depending on the timing of incoming dopamine action potentials relative to cholinergic neurons (116). Differences in these local interactions will result in region-specific dopamine influence.

(3) The presynaptic influence of other neurotransmitters on dopamine release may result in dopamine release without involving changes in action potential frequency (117), which is also apparent from behavioral

tests (118). Thus, some behavioral dopamine functions may not require action potential changes at dopamine cell bodies.

(4) As soon as dopamine is released into the extracellular space, the dopamine transporter recaptures the intact molecule within tens to hundreds of milliseconds into the dopamine neurons and thus limits the dopamine action on postsynaptic receptors (119). The known local variations in dopamine transporter concentration result in regional differences of dopamine level. For example, dopamine transporter concentration is less dense in nucleus accumbens as compared to dorsal striatum (120). With different recapture speed, postsynaptic dopamine action follows different time courses in different brain regions.

(5) The impact of dopamine neurotransmission on postsynaptic neurons depends on the type of postsynaptic dopamine receptor. Striatal neurons carrying D1 or D2 types of dopamine receptor have opposite action on striatal neurons and project to different nuclei of the basal ganglia (121). These distinct striatal neurons affect learning and choices in opposite ways (122-124). While it is unknown whether these effects of striatal neurons derive from their dopamine inputs, the existence of dopamine receptors on these neurons make the efferent routing of dopamine influences at least possible.

(6) Dopamine neurotransmission enhances the synaptic weights from recently active inputs onto postsynaptic striatal and cortical neurons while leaving inactive synapses unchanged, thus following the notion of a three-factor Hebbian learning mechanism based on the anatomical triad of cortical inputs, local striatal or cortical neurons, and dopamine varicosities (125-129). These changes require D1 and D2 dopamine receptors (130, 131) and occur at dendritic spines within a time window compatible with behavioral conditioning (132). These plasticity effects may be key mechanisms by which the dopamine RPE signal mediates RL.

Thus, while allowing temporally and spatially precise measurements, the action potentials of midbrain dopamine neurons carry only the first step of RPE information; subsequent steps are likely to modulate the influence of the RPE signal depending on other presynaptic inputs and according to the specificity of postsynaptic neurons. While these multiple modulations are important to take into account, they do not change fundamentally the concepts of dopamine influence on learning, as attested by the reward-related behavioral effects of optogenetic excitation and inhibition of midbrain dopamine cell bodies and striatal dopamine axons.

SI References

1. Berke JD. What does dopamine mean? Nat Neurosci 21: 787-793, 2018.
2. Cox J, Witten IB. Striatal circuits for reward learning and decision-making. Nat Rev Neurosci 20: 482-494, 2019.
3. Schultz W. Multiple dopamine functions at different time courses. Ann Rev Neurosci 30: 259-288, 2007.
4. Schultz W. Responses of midbrain dopamine neurons to behavioral trigger stimuli in the monkey. J Neurophysiol 56: 1439-1462, 1986.
5. Romo R, Schultz W. Dopamine neurons of the monkey midbrain: Contingencies of responses to active touch during self-initiated arm movements. J. Neurophysiol. 63: 592-606, 1990.
6. Schultz W. Recent advances in understanding the role of phasic dopamine activity. F1000Research 8: 1680, 2019.
7. Schultz W. Dopamine reward prediction error signalling: a two-component response. Nat Rev Neurosci 17: 183-195, 2016.
8. Schultz W, Ruffieux A, Aebischer P. The activity of pars compacta neurons of the monkey substantia nigra in relation to motor activation. Exp. Brain Res. 51: 377-387, 1983.
9. Phillips PEM, Stuber GD, Heien MLAV, Wightman RM, Carelli RM. Subsecond dopamine release promotes cocaine seeking. Nature 422: 614-618, 2003.
10. Howe MW, Tierney PL, Sandberg SG, Phillips, PEM, Graybiel AM. Prolonged dopamine signalling in striatum signals proximity and value of distant rewards. Nature 500: 575-579, 2013.
11. Dodson, PD, Jakob K. Dreyer JK, Jennings KA, Syeda ECJ, Wade-Martins R, Cragg SJ, Bolam JP, Magill PJ. Representation of spontaneous movement by dopaminergic neurons is cell-type selective and disrupted in parkinsonism. Proc Natl Acad Sci USA 113: E2180-E2188, 2016.
12. Engelhard B, Finkelstein J, Cox J, Fleming W, Jang HJ, Ornelas S, Koay SA, Thiberge SY, Daw ND, Tank DW, Witten IB. Specialized coding of sensory, motor and cognitive variables in VTA dopamine neurons. Nature 570: 509-513, 2019.
13. Musall S, Kaufman MT, Juavinett AL, Gluf S, Churchland AK. Single-trial neural dynamics are dominated by richly varied movements. Nat Neurosci 22: 1677-1686, 2019.
14. Tremblay S, Testard C, DiTullio RW, Inchauspé J, Petrides M. Neural cognitive signals during spontaneous movements in the macaque. Nat Neurosci 26: 295–305, 2023.
15. DeLong MR, Crutcher MD, Georgopoulos AP. Relations between movement and single cell discharge in the substantia nigra of the behaving monkey. J Neurosci 3: 1599-1606, 1983.

16. Schultz W, Romo R. Dopamine neurons of the monkey midbrain: Contingencies of responses to stimuli eliciting immediate behavioral reactions. J Neurophysiol 63: 607-624, 1990.
17. Ljungberg T, Apicella P, Schultz W. Responses of monkey dopamine neurons during learning of behavioral reactions. J Neurophysiol 67: 145-163, 1992.
18. Nomoto K, Schultz W, Watanabe T, Sakagami M. Temporally extended dopamine responses to perceptually demanding reward-predictive stimuli. J Neurosci 30: 10692–10702, 2010.
19. Fiorillo CD, Song MR, Yun SR. Multiphasic temporal dynamics in responses of midbrain dopamine neurons to appetitive and aversive stimuli. J Neurosci 33: 4710–4725, 2013.
20. Eshel N, Tian J, Bukwich M, Uchida N: Dopamine neurons share common response function for reward prediction error. Nat Neurosci 19: 479-486, 2016.
21. Schultz W. Dopamine reward prediction error signalling: a two-component response. Nat Rev Neurosci 17: 183-195, 2016.
22. Sutton RS, Barto AG. Reinforcement Learning. Cambridge, MA: MIT Press, 1998.
23. Mirenowicz J, Schultz W. Preferential activation of midbrain dopamine neurons by appetitive rather than aversive stimuli. Nature 379: 449-451, 1996.
24. Kobayashi S, Schultz W. Reward contexts extend dopamine signals to unrewarded stimuli. Curr Biol 24: 56-62, 2014.
25. Matsumoto H, Tian J, Uchida N, Watabe-Uchida M. Midbrain dopamine neurons signal aversion in a reward-context-dependent manner. eLife 5: e17328, 2016.
26. Lak A, Stauffer WR, Schultz W. Dopamine neurons learn relative chosen value from probabilistic rewards. eLife 5: e18044, 2016.
27. Menegas W, Babayan B., Uchida N, Watabe-Uchida M. Opposite initialization to novel cues in dopamine signaling in ventral and posterior striatum in mice. eLife 6, e21886, 2017.
28. Takahashi YK, Batchelor HM, Liu B, Khanna A, Morales M, Schoenbaum G. Dopamine neurons respond to errors in the prediction of sensory features of expected rewards. Neuron 95: 1395–1405, 2017.
29. de Jong JW, Afjei SA, Dorocic IP, Peck JR, Liu C, Kim CK, Tian L, Karl Deisseroth K, Lammel S. A neural circuit mechanism for encoding aversive stimuli in the mesolimbic dopamine system. Neuron 101: 133–151, 2019.
30. Kutlu MG, Zachry JE, Melugin PR, Cajigas SA, Chevee MF, Kelley SJ, Kutlu B, Tian L, Siciliano CA, Calipari ES. Dopamine release in the nucleus accumbens core signals perceived saliency. Curr Biol 31: 4748-4761, 2021.
31. Menegas W, Akiti K, Amo R, Uchida N, Watabe-Uchida N. Dopamine neurons projecting to the posterior striatum reinforce avoidance of threatening stimuli. Nat Neurosci 21: 1421-1430, 2018.
32. Matsumoto M, Hikosaka O. Two types of dopamine neuron distinctively convey positive and negative motivational signals. Nature 459: 837-841, 2009.
33. Oleson EB, Gentry RN, Chioma VC, Cheer JF. Subsecond dopamine release in the nucleus accumbens predicts conditioned punishment and its successful avoidance. J Neurosci 32: 14804-14808, 2012.
34. Satoh T, Nakai S, Sato T, Kimura, M. Correlated coding of motivation and outcome of decision by dopamine neurons. J Neurosci 23: 9913-9923, 2003.
35. Lak A, Stauffer WR, Schultz W. Dopamine prediction error responses integrate subjective value from different reward dimensions. Proc Natl Acad Sci (USA) 111: 2343-2348, 2014.
36. Fiorillo CD, Tobler PN, Schultz W. Discrete coding of reward probability and uncertainty by dopamine neurons. Science 299: 1898-1902, 2003.
37. Phillips PEM, Stuber GD, Heien MLAV, Wightman RM, Carelli RM. Subsecond dopamine release promotes cocaine seeking. Nature 422: 614-618, 2003
38. Jin X, Costa RM. Start/stop signals emerge in nigrostriatal circuits during sequence learning. Nature 466: 457-462, 2010.
39. Dodson, PD, Jakob K. Dreyer JK, Jennings KA, Syeda ECJ, Wade-Martins R, Cragg SJ, Bolam JP, Magill PJ. Representation of spontaneous movement by dopaminergic neurons is cell-type selective and disrupted in parkinsonism. Proc Natl Acad Sci USA 113: E2180-E2188, 2016.
40. Howe MW, Dombeck DA. Rapid signalling in distinct dopaminergic axons during locomotion and reward. Nature 535: 505-510, 2016.
41. Parker NF, Cameron CM, Taliaferro JP, Lee J, Choi JY, Davidson TJ, Daw ND, Witten IB. Reward and choice encoding in terminals of midbrain dopamine neurons depends on striatal target. Nat Neurosci 19: 845-854, 2016.
42. Alves da Silva J, Tecuapetla F, Paixão V, Costa RM. Dopamine neuron activity before action initiation gates and invigorates future movements. Nature 554: 244-248, 2018.
43. Coddington LT, Dudman JT. Learning from action: reconsidering movement signaling in midbrain dopamine neuron activity. Neuron 104: 63-77, 2019.

44. Wassum KM, Ostlund SB, Maidment NT. Phasic meso-limbic dopamine signaling precedes and predicts performance of a self-initiated action sequence task. Biol Psychiat 71: 846–854, 2012.
45. Collins AL, Greenfield VY, Bye JK, Linker KE, Wang AS, Wassum KM. Dynamic mesolimbic dopamine signaling during action sequence learning and expectation violation. Sci Rep 6: 20231, 2016.
46. Hamid AA, Pettibone JR, Mabrouk OS, Hetrick VL, Schmidt R, Vander Weele CM, Kennedy RT, Aragona BJ, Berke JD. Mesolimbic dopamine signals the value of work. Nat Neurosci 19: 117-126, 2016.
47. Hamilos AE, Spedicato G, Hong Y, Sun F, Li Y, Assad JA. Dynamic dopaminergic activity controls the timing of self-timed movement. bioRxiv 2020.05.13.094904, 2020.
48. Stuber GD, Klanker M, de Ridder B, Bowers MS, Joosten RN, Feenstra MG, Bonci A. Reward-predictive cues enhance excitatory synaptic strength onto midbrain dopamine neurons. Science 321: 1690–1692, 2008.
49. Flagel SB, Clark JJ, Robinson TE, Mayo L, Czuj A, Willuhn I, Akers CA, Clinton SM, Phillips PE, Akil H: A selective role for dopamine in stimulus-reward learning. Nature 469: 53-57, 2011.
50. Cohen JY, Haesler S, Vong L, Lowell BB, Uchida N. Neuron-type-specific signals for reward and punishment in the ventral tegmental area. Nature 482: 85-88, 2012.
51. Hart AS, Rutledge RB, Glimcher PW, Phillips PEM. Phasic dopamine release in the rat nucleus accumbens symmetrically encodes a reward prediction error term. J Neurosci 34: 698-704, 2014.
52. Eshel N, Bukwich M, Rao V, Hemmelder V, Tian J, Uchida N: Arithmetic and local circuitry underlying dopamine prediction errors. Nature 525, 243–246, 2015.
53. Menegas W, Bergan JF, Ogawa SK, Isogai Y, Umadevi Venkataraju K, Osten P, Uchida N, Watabe-Uchida M. Dopamine neurons projecting to the posterior striatum form an anatomically distinct subclass. eLife 4, e10032, 2015.
54. Babayan BM, Uchida N, Gershman SJ. Belief state representation in the dopamine system. Nat Comm 9: 1891, 2018.
55. Schultz W, Dayan P, Montague RR. A neural substrate of prediction and reward. Science 275: 1593-1599, 1997.
56. Gershman SJ. Dopamine ramps are a consequence of reward prediction errors. Neural Computation 26, 467–471, 2014.
57. Lloyd K, Dayan P. Tamping ramping: algorithmic, implementational, and computational explanations of phasic dopamine signals in the accumbens. PLoS Comput Biol 11, e1004622, 2015.
58. de Lafuente O, Romo R. Dopamine neurons code subjective sensory experience and uncertainty of perceptual decisions. Proc Natl Acad Sci 108: 19767-19771, 2011.
59. Suri R, Schultz W. A neural network with dopamine-like reinforcement signal that learns a spatial delayed response task. Neuroscience 91: 871-890, 1999.
60. Amo R, Matias S, Yamanak A, Tanaka KF, Uchida N, Watabe-Uchida M. A gradual shift of dopamine responses mirrors the progression of temporal difference error in machine learning. Nat Neurosci 25: 1082-1092, 2022.
61. Mikhael JG, Kim HR, Uchida N, Gershman SJ. The role of state uncertainty in the dynamics of dopamine. Curr Biol 32: 1077-1087, 2022.
62. Kobayashi S, Schultz W. Influence of reward delays on responses of dopamine neurons. J Neurosci 28: 7837-7846, 2008.
63. Paton JJ, Belova MA, Morrison SE, Salzman CD. The primate amygdala represents the positive and negative value of visual stimuli during learning. Nature 439: 865-870, 2006.
64. Gründemann J, Bitterman Y, Lu T, Krabbe S, Grewe BF, Schnitzer MJ, Lüthi A. Amygdala ensembles encode behavioral states. Science 364: eaav8736, 2019.
65. Grabenhorst F, Hernadi I, Schultz W. Prediction of economic choice by primate amygdala neurons. Proc Natl Acad Sci (USA) 109: 18950-18955, 2012.
66. Jarzebowski P, Hay YA, Benjamin F. Grewe, Paulsen O. Different encoding of reward location in dorsal and intermediate hippocampus. Curr Biol 32: 834-841, 2022.
67. Carta I, Chen CH, Schott AL, Dorizan S, Khodakhah K. Cerebellar modulation of the reward circuitry and social behavior. Science 363: eaav0581 (2019).
68. Rigotti M, Barak O, Warden MR, Wang X-J, Daw ND, Miller EK, Fusi S. The importance of mixed selectivity in complex cognitive tasks. Nature 497: 585-590, 2013.
69. Shuler, MG, Bear MF. Reward timing in the primary visual cortex. Science 311: 1606-1609, 2006.
70. Stringer C, Pachitariu M, Steinmetz N, Reddy CB, Carandini M, Harris KD. Spontaneous behaviors drive multidimensional, brainwide activity. Science 364: eaav7893, 2019.
71. Wannier TMJ, Maier MA, Hepp-Reymond MC. Responses of motor cortex neurons to visual stimulation in the alert monkey. Neurosci Lett 98: 63-68, 1989.
72. Hatsopoulos NG, Suminski AJ. Sensing with the motor cortex. Neuron 72: 477-487, 2011.

73. Luce RD. Individual Choice Behavior: A Theoretical Analysis. New York: Wiley, 1959.
74. von Neumann J, Morgenstern O. *The Theory of Games and Economic Behavior*. Princeton: Princeton University Press, 1944.
75. Marschak J. Rational behavior, uncertain prospects, and measurable utility. Econometrica 18: 111-141, 1950.
76. Savage LJ. *The Foundations of Statistics*. New York: Wiley, 1954.
77. Stauffer WR, Lak A, Schultz W. Dopamine reward prediction error responses reflect marginal utility. Curr Biol 24: 2491-2500, 2014.
78. Genest W, Stauffer WR, Schultz W. Utility functions predict variance and skewness risk preferences in monkeys. Proc Natl Acad Sci (USA) 113: 8402-8407, 2016.
79. Caraco T, Martindale S, Whitham TS. An empirical demonstration of risk-sensitive foraging preferences. Anim Behav 28: 820-830, 1980.
80. Machina MJ. Choice under uncertainty: problems solved and unsolved. J Econ Perspect 1: 121-154, 1987.
81. Ferrari-Toniolo S, Bujold P, Schultz W. Probability distortion depends on choice sequence in rhesus monkeys. J Neurosci 39: 2915-2929, 2019.
82. Chen X, Stuphorn V. Inactivation of medial frontal cortex changes risk preference. Curr Biol 28: 3114-3122, 2018.
83. Weber BJ, Chapman GB. Playing for peanuts: Why is risk seeking more common for low-stakes gambles? Organiz Behav Human Dec Proc 97: 31–46, 2005.
84. Markowitz H. The utility of wealth. J Polit Econ 6: 151-158, 1952.
85. McFadden D. Conditional logit analysis of qualitative choice behavior. In: Frontiers in Econometrics, chapter 4:105–142 (Academic Press, New York), 1974.
86. McFadden D. Economic Choices. Am Econ Rev 91:351–378, 2001.
87. Bujold PM, Ferrari-Toniolo S, Chi U Seak L, Schultz W. Comparing utility functions between risky and riskless choice in rhesus monkeys. Anim Cog 25: 385-399, 2022.
88. Yamada H, Tymula A, Louie K, Glimcher PW. Thirst-dependent risk preferences in monkeys identify a primitive form of wealth. Proc Natl Acad Sci (USA) 110: 15788-15793, 2013.
89. Ferrari-Toniolo S, Bujold P, Schultz W. Non-human primates satisfy utility maximization in compliance with the continuity axiom of Expected Utility Theory. J Neurosci 41: 2964-2979, 2021.
90. Ferrari-Toniolo S, Seak LCU, Schultz W. Risky choice: probability weighting explains Independence Axiom violations in monkeys. J Risk Uncertain 10.1007: s11166-022-09388-7, 2022.
91. Allais M. Le comportement de l'homme rationnel devant le risque: Critique des postulats et axiomes de l'ecole Americaine. Econometrica 21: 503-546, 1953.
92. Blavatskyy P, Ortmann A, Panchenko V. On the experimental robustness of the Allais Paradox. Am Econ J: Microecon 14: 143-163, 2022.
93. Schultz W, Stauffer WR, Lak A, Pastor-Bernier A. Smarter than humans: rationality reflected in primate neuronal reward signals. Curr Op Behav Sci 41: 51-56, 2021.
94. Kahneman D, Tversky A. Prospect theory: an analysis of decision under risk. *Econometrica* 47: 263–291, 1979.
95. Stauffer WR, Lak A, Bossaerts P, Schultz W. Economic choices reveal probability distortion in monkeys. J Neurosci 35: 3146-3154, 2015.
96. Bujold PM, Ferrari-Toniolo S, Schultz W. Adaptation of utility functions to reward distribution in rhesus monkeys. Cognition 214: 104764, 2021.
97. Raghuraman AP, Padoa-Schioppa C. Integration of multiple determinants in the neuronal computation of economic values. J Neurosci 34: 11583-11603, 2014.
98. Rothschild M, Stiglitz JE. Increasing risk: I. A definition. J Econ Theory 2: 225-243, 1970.
99. Mas-Colell A, Whinston M, Green J. Microeconomic Theory. New York: Oxford Univ Press, 1995.
100. Corbett D, Wise RA. Intracranial self-stimulation in relation to the ascending dopaminergic systems of the midbrain: A moveable microelectrode study. Brain Res 185: 1-15, 1980.
101. Tsai H-C, Zhang F, Adamantidis A, Stuber GD, Bonci A, de Lecea L, Deisseroth K. Phasic firing in dopaminergic neurons is sufficient for behavioral conditioning. Science 324: 1080-1084, 2009.
102. Tan KR, Yvon C, Turiault M, Mirzabekov JJ, Doehner J, Labouèbe G, Deisseroth K, Tye KM, Lüscher C. GABA neurons of the VTA drive conditioned place aversion. Neuron 73: 1173-1183, 2012.
103. Stauffer WR, Lak A, Yang A, Borel M, Paulsen O, Boyden E, Schultz W. Dopamine neuron-specific optogenetic stimulation in Rhesus macaques. Cell 166: 1564-1571, 2016.
104. Tesauro G. TD-Gammon, a self-teaching backgammon program, achieves master-level play. Neural Comp 6: 215-219, 1994.

105. Mnih V, Kavukcuoglu K, Silver D, Rusu AA, Veness J, Bellemare MG, Graves A, Riedmiller M, Fidjeland AK, Ostrovski G, Petersen S, Beattie C, Sadik A, Antonoglou I, King H, Kumaran D, Wierstra D, Legg S, Hassabis D. Human-level control through deep reinforcement learning. Nature 518: 529-533, 2015.
106. Silver D, Schrittwieser J, Simonyan K, Antonoglou I, Huang A, Guez A, Hubert T, Baker L, Lai M, Bolton A, Chen Y, Lillicrap T, Hui F, Sifre L, van den Driessche G, Graepel T, Hassabis D. Mastering the game of Go without human knowledge. Nature 550: 354-359, 2017.
107. Silver D, Hubert T, Schrittwieser J, Antonoglou I, Lai M, Guez A, Lanctot M, Sifre L, Kumaran D, Graepel T, Lillicrap T, Simonyan K, Hassabis D. A general reinforcement learning algorithm that masters chess, shogi, and Go through self-play. Science 362: 1140-1144, 2018.
108. Purves D. What does AI's success playing complex board games tell brain scientists? Proc Natl Acad Sci USA 116: 14785-14787, 2019.
109. Matsuda W, Furuta T, Nakamura KC, Hioki H, Fujiyama F, Arai R, Kaneko T. Single nigrostriatal dopaminergic neurons form widely spread and highly dense axonal arborizations in the neostriatum. J Neurosci 29:444-453, 2009.
110. Swadlow HA, Kocsis JD, Waxman SG. Modulation of impulse conduction along the axonal tree. Ann Rev Biophys Bioeng 9: 143-179, 1980.
111. Debanne D. Information processing in the axon. Nat Rev Neurosci 5: 304-316, 2004.
112. Wightman RM, Heien ML, Wassum KM, Sombers LA, Aragona BJ, Khan AS, Ariansen JL, Cheer JF, Phillips PE, Carelli RM: Dopamine release is heterogeneous within microenvironments of the rat nucleus accumbens. Eur J Neurosci 26: 2046-2054, 2007.
113. Gonon FG, Buda MJ. Regulation of dopamine release by impulse flow and by autoreceptors as studied by in vivo voltammetry in the rat striatum. Neuroscience 14: 765-774, 1985.
114. Kita JM, Parker LE, Phillips PEM, Garris PA, Wightman RM. Paradoxical modulation of short-term facilitation of dopamine release by dopamine autoreceptors. J Neurochem 102: 1115-1124, 2007.
115. Chesselet MF. Presynaptic regulation of neurotransmitter release in the brain: Facts and hypothesis. Neuroscience 12: 347-375, 1984.
116. Threlfell S, Lalic T, Platt NJ, Jennings KA, Deisseroth K, Cragg SJ. Striatal dopamine release is triggered by synchronized activity in cholinergic interneurons. Neuron 75: 58–64, 2012.
117. Giorguieff MF, Le Floch ML, Glowinski J, Besson JM. Involvement of cholinergic presynaptic receptors of nicotinic and muscarinic types in the control of the spontaneous release of dopamine from striatal dopaminergic terminals in the rat. J Pharmacol exp Ther 200: 535-544, 1977.
118. Mohebi A, Pettibone JR, Hamid AA, Wong J-MT, Vinson LT, Patriarchi T, Tian L, Kennedy RT, Berke JD. Dissociable dopamine dynamics for learning and motivation. Nature 570: 65-70, 2019.
119. Kawagoe KT, Garris PA, Wiedemann DJ, Wightman RM. Regulation of transient dopamine concentration gradients in the microenvironment surrounding nerve terminals in the rat striatum. Neuroscience 51: 55-64, 1992.
120. Coulter CL, Happe HK, Murrin LC (1996) Postnatal development of the dopamine transporter: a quantitative autoradiographic study. Brain Res Dev Brain Res 92:172–181.
121. Gerfen CR. The neostriatal mosaic: multiple levels of comportmental organization. Trends Neurosci. 15: 133-139, 1992.
122. Kravitz AV, Tye LD, Kreitzer AC. Distinct roles for direct and indirect pathway striatal neurons in reinforcement. Nat Neurosci 15: 816-818, 2012.
123. Tai L-H, Lee AM, Benavidez N, Bonci A, Wilbrecht L. Transient stimulation of distinct subpopulations of striatal neurons mimics changes in action value. Nat Neurosci 15: 1281-1289, 2012.
124. Yttri EA, Dudman JT. Opponent and bidirectional control of movement velocity in the basal ganglia. Nature 533: 402-406, 2016.
125. Freund TF, Powell JF, Smith AD. Tyrosine hydroxylase-immunoreactive boutons in synaptic contact with identified striatonigral neurons, with particular reference to dendritic spines. Neuroscience 13: 1189-1215, 1984.
126. Schultz W. Predictive reward signal of dopamine neurons. J. Neurophysiol. 80: 1-27, 1998
127. Reynolds JNJ, Hyland BI, Wickens JR. A cellular mechanism of reward-related learning. Nature 413: 67-70, 2001.
128. Matsuda Y, Marzo A, Otani S. The presence of background dopamine signal converts long-term synaptic depression to potentiation in rat prefrontal cortex. J Neurosci 26: 4803– 4810, 2006.
129. Xu TX, Yao WD. D1 and D2 dopamine receptors in separate circuits cooperate to drive associative long-term potentiation in the prefrontal cortex. Proc Natl Acad Sci USA 107: 16366–16371, 2010.
130. Pawlak V, Kerr JND. Dopamine receptor activation is required for corticostriatal spike-timing-dependent plasticity. J Neurosci 28: 2435–2446, 2008.

131. Shen W, Flajolet M, Greengard P, Surmeier DJ. Dichotomous dopaminergic control of striatal synaptic plasticity. Science 321: 848-851, 2008.
132. Yagishita S, Hayashi-Takagi A, Ellis-Davies GCR, Urakubo H, Ishii S, Kasai H. A critical time window for dopamine actions on the structural plasticity of dendritic spines. Science 345: 1616-1620, 2014.
